# Magnetic Force-Based Microfluidic Techniques for Cellular and Tissue Bioengineering

**DOI:** 10.3389/fbioe.2018.00192

**Published:** 2018-12-19

**Authors:** Sena Yaman, Muge Anil-Inevi, Engin Ozcivici, H. Cumhur Tekin

**Affiliations:** Department of Bioengineering, Izmir Institute of Technology, Izmir, Turkey

**Keywords:** magnetic manipulations, microfluidics, rare cell separation, cell culture, tissue engineering

## Abstract

Live cell manipulation is an important biotechnological tool for cellular and tissue level bioengineering applications due to its capacity for guiding cells for separation, isolation, concentration, and patterning. Magnetic force-based cell manipulation methods offer several advantages, such as low adverse effects on cell viability and low interference with the cellular environment. Furthermore, magnetic-based operations can be readily combined with microfluidic principles by precisely allowing control over the spatiotemporal distribution of physical and chemical factors for cell manipulation. In this review, we present recent applications of magnetic force-based cell manipulation in cellular and tissue bioengineering with an emphasis on applications with microfluidic components. Following an introduction of the theoretical background of magnetic manipulation, components of magnetic force-based cell manipulation systems are described. Thereafter, different applications, including separation of certain cell fractions, enrichment of rare cells, and guidance of cells into specific macro- or micro-arrangements to mimic natural cell organization and function, are explained. Finally, we discuss the current challenges and limitations of magnetic cell manipulation technologies in microfluidic devices with an outlook on future developments in the field.

## Introduction

The manipulation of living cells by an external stimulus is an important tool for separation or detection of cells of interest and to guide cells in tissue engineering applications. Together with recent advances in engineering and technology, systems and designs varying in size, shape, complexity, and cost have been developed for various cell manipulation applications. These applications include blood cell separation (Ji et al., 2008), rare cell (e.g., circulating tumor cells) isolation in blood (Huang et al., 2013; Karabacak et al., 2014), detection of pathogens (Ho, 2014; Huang et al., 2017), cell counting for disease monitoring (e.g. CD4^+^ T cell counting for HIV progression) (Cheng et al., 2007; Boyle et al., 2012), stem cell enrichment (Stephens et al., 1996; Muslimov et al., 2017) and organization of cells into designed spatial arrangements in two or three dimensional cultures (Ger et al., 2013; Tseng et al., 2014).

Most cell manipulation techniques are based on physical and/or affinity-based approaches. Physical manipulation techniques are driven by intrinsic cell properties (e.g., deformability, density, electrical capacitance, or resistance, size, magnetic susceptibility, mass, morphology) while affinity-based techniques use “labels” (e.g., particle-antibody conjugates specific to a membrane protein) to manipulate cells of interest. Operation principles include electrical (Fuhr et al., 1994; Voldman, 2006; CemaŽar et al., 2016), mechanical (Lo et al., 2000; Chronis and Lee, 2005; Kim et al., 2008b), affinity (Jin et al., 2009), acoustic (Coakley et al., 1989; Laurell et al., 2007; Lenshof et al., 2017), optical (Ashkin et al., 1987; Grier, 2003; Cheng et al., 2017), and magnetic (Pamme and Manz, 2004; Pamme, 2006; Zhao et al., 2016) forces or combined application of these factors (Wiklund et al., 2006).

The basic principle of magnetic force-based manipulation relies on a magnetic field strength, field gradient, and a magnetic susceptibility difference between the cell of interest and the surrounding environment (Data Sheet 1 in the Supplementary Material). This strategy offers several advantages compared to its alternatives. First, the non-contact nature of the technique minimizes potential hazardous effects that could reduce cell viability/integrity (Morais et al., 2006). Second, the generation of a magnetic field does not depend on complex or expensive instrumentation as it can be created with an externally located, simple, low-cost rare earth magnet (Zeng et al., 2013; Durmus et al., 2015). Third, magnetic manipulation has low sensitivity to internal and external factors such as ionic strength, surface charges, pH and temperature (Nguyen, 2012).

Movement of cells under a magnetic field is usually referred to as magnetophoresis which can be performed in two ways. Cells are migrated to a region of high magnetic field strength (positive magnetophoresis) or escape from such a region (negative magnetophoresis). Since most cells are not inherently magnetic in nature, researchers generally exploit extrinsic magnetic properties for positive magnetophoresis by labeling cells with magnetic nanoparticles. In bio-separation applications, magnetic nanoparticles (MNPs) are commonly preferred as labels due to their unique magnetic properties, large surface to volume ratio, ability to selectively bind to target cell with the recognition ligands coupled onto their surface, and biocompatibility (Jiang et al., 2004; Gu et al., 2006; Issa et al., 2013). In terms of cell separation, magnetically-activated cell sorting (MACS) is a widely accepted technique in which MNPs decorated with cell membrane antigen-specific antibodies are used (Thiel et al., 1998). Although most sorting techniques rely on labeling cells with MNPs via cell surface antigens, it is also possible to contrast cells that contain different magnetic bead distributions based on their endocytotic capacity such as monocytes and macrophages (Robert et al., 2011). In addition, intrinsic magnetic properties of erythrocytes with iron-containing hemoglobin have been exploited in positive magnetophoresis for cell separation (Zborowski et al., 2003).

Positive magnetophoresis is a useful tool in the field of two and three dimensional (2D and 3D) cell culture and can be used to assemble cells into 3D cellular spheroids as building blocks (Mattix et al., 2014a; Parfenov et al., 2018), to pattern cells in culture for a suitable cellular microenvironment (Ino et al., 2009; Whatley et al., 2014), to guide cells into sheet-like structures for close cellular contact (Ito et al., 2004b; Ishii et al., 2014) and to enhance the seeding efficiency of cells into scaffolds in tissue engineering applications (Thevenot et al., 2008). However, since positive magnetophoresis heavily rely on the labeling of cells with magnetic particles, problems related to adequate and standard cellular internalization (Küstermann et al., 2008; Wildgruber et al., 2009), time consuming experimental steps (Chen et al., 2013; Calero et al., 2014) and possible biological interference of magnetic labels (Kostura et al., 2004; Peyman et al., 2009; Kedziorek et al., 2010) stand out as the most common limiting factors.

In recent years, a label-free magnetic manipulation alternative based on negative magnetophoresis has been developed to eliminate the adverse effects of cell labeling. In this method, cells are placed in a medium containing either a paramagnetic salt solution (Peyman et al., 2009) or a ferrofluid (Zhu et al., 2010). Since cells magnetize less than the medium, cells are focused in the lower magnetic field regions when placed under a magnetic field (Shen et al., 2012). Thereby, cells can be manipulated based on the arranged magnetic field pattern. This method was successfully used to trap bacteria (Wang et al., 2016b), to separate tumor cells (Zhao et al., 2017a,b), and to detect lipid-accumulating bone marrow cells (Sarigil et al., 2018) and cells with impaired function (e.g., sickle cells) (Knowlton et al., 2015). Moreover, there are successful applications of this technique to create ordered cellular structures such as the assembly of cells into linear arrangements (Krebs et al., 2009) or spheroids (Akiyama and Morishima, 2011a) and levitation of cell encapsulated polymers (Tasoglu et al., 2015b).

The motivation of this review is to address the fundamental principles, advantages, and challenges of recent studies applying labeled or label-free magnetic force-based techniques with state-of-the-art design or technology for cell and tissue manipulation. This review discusses the major applications of *in vitro* magnetophoresis from a cellular and tissue bioengineering perspective, namely, 1) rare cell separation, and 2) 2D and 3D cell culture.

## Review of Magnetic Manipulation Applications

The importance of efficient *in vitro* cell detection and sorting platforms has increased in parallel with the growing demand for the diagnosis of cancer and infectious diseases, enrichment of rare cells, and monitoring of environmental safety and public health (Mairhofer et al., 2009; Pratt et al., 2011; Chen et al., 2012; Foudeh et al., 2012). Consequently, a variety of magnetic cell sorting and detection methods and devices have been developed over the past few decades. Besides *in vitro* sorting and detection, the magnetic guidance of cells has been exploited in the organization of cells to mimic natural cell arrangements and functions. Magnetic cell manipulation methods are useful tools to form 3D cellular assemblies, to guide single cells or 3D building blocks into a desired pattern, to create cell sheets with tight cellular contacts and to enhance cell seeding efficiency into scaffolds. Lately, the combination of magnetism and microfluidic concepts, which is termed “magnetofluidics” (Lenshof and Laurell, 2010; Nguyen, 2012; Hejazian and Nguyen, 2016) has advanced rapidly due to several advantages: (1) an external magnetic force can be created with a simple, small-sized permanent magnet (Hejazian and Nguyen, 2016), (2) micro- or nano-sized magnetic labels can be readily used for manipulating biological components inside microfluidic channels (Kwak et al., 2017), (3) magnetofluidics enables continuous-flow separation of cells (e.g., continuous separation of erythrocytes and leukocytes from the whole blood) (Pamme and Wilhelm, 2006) and (4) the magnetic field can pass through various components of microfluidic systems such as glass, metals, plastics, and liquids, which allows contactless manipulation of cells (Bhuvanendran Nair Gourikutty et al., 2016b). Considering the growing trend, the following part of the review focuses on the recent advancements and challenges in magnetofluidic detection, sorting and cell culture.

### Rare Cell Screening: Isolation and Enrichment of Rare Cells

Rare cells are defined as those which are present at fewer than 1,000 cells in 1 mL of sample (Dharmasiri et al., 2010) such as clinically important stem cells (e.g., hematopoietic stem cells) and circulating tumor cells (CTCs) (Chen et al., 2014). CTC detection and isolation techniques have opened a new era in cancer prognosis and development of personalized chemotherapy or radiotherapy (Greene et al., 2012; Toss et al., 2014). CTC-derived organoid cultures have potential applications in disease modeling with a structure that more closely resembles natural organ systems compared to 2D cell cultures (Boj et al., 2015). Stem cells (SCs), on the other hand, are promising candidates for regenerative medicine. They are isolated and reinjected to promote natural repair mechanisms in the body (Sasaki et al., 2008). In fact, cell regeneration approaches for the treatment of several diseases and disorders such as cardiac, neurodegenerative, kidney, and lung diseases are under clinical investigation (Chen and Hou, 2016; Mathur et al., 2016; Kumar et al., 2017; Li et al., 2017). Given that tumor and stem cells have great therapeutic and regenerative potential, there is a crucial need for developing efficient detection and isolation methods for pure and transferable rare cell populations.

**Figure 1:**
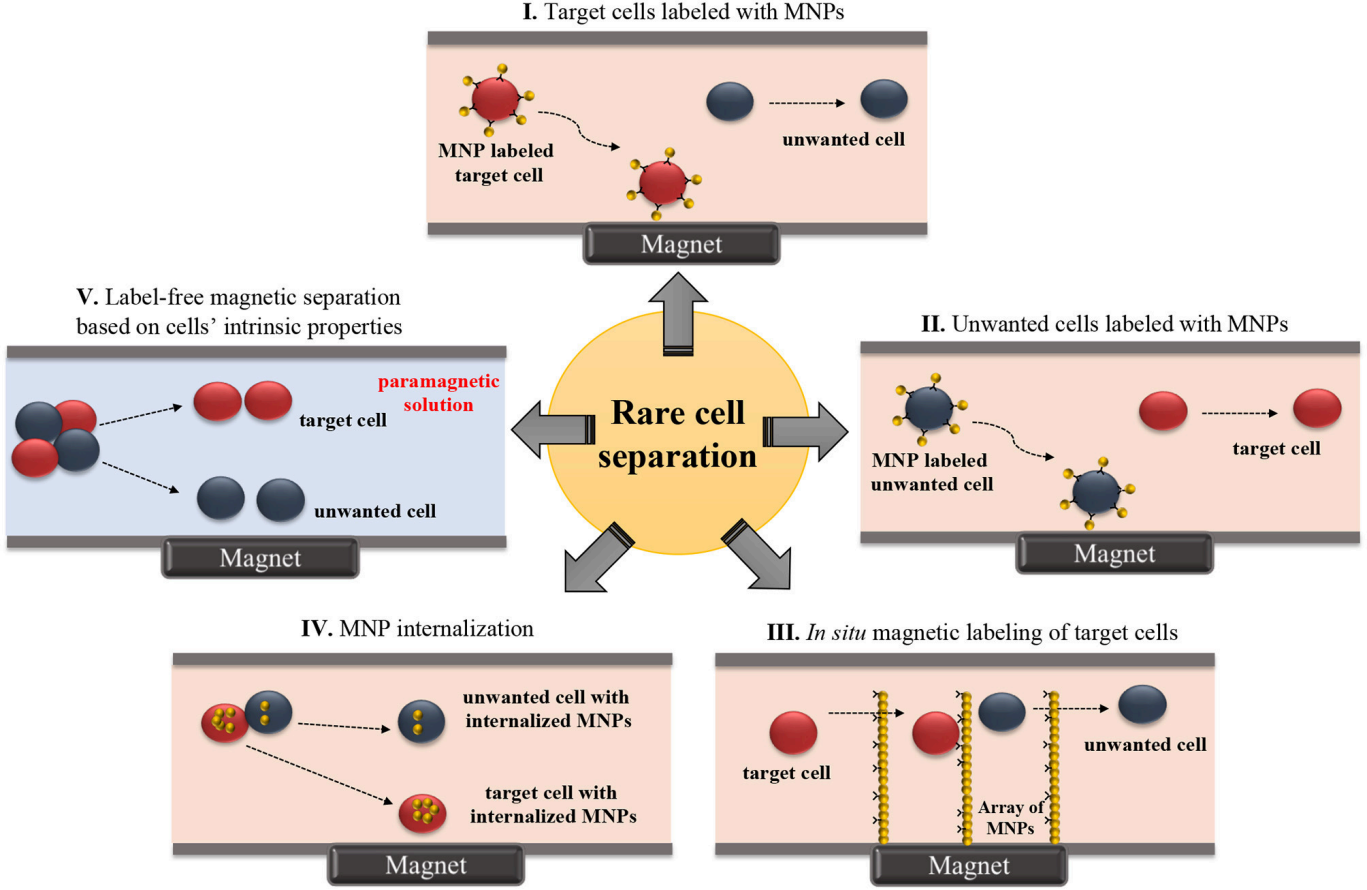
Schematic illustration of microfluidic-based rare cell isolation methods. **(I)** Target cells labeled with MNPs, **(II)** Unwanted cells labeled with MNPs, **(III)** *In situ* magnetic labeling of target cells, **(IV)** MNP internalization, and **(V)** Label-free magnetic separation based on cells' intrinsic properties.

Most magnetic rare cell separation methods depend on targeting surface antigens on cells using antibody coupled-magnetic labels (Figure 1 and Table 1). On the other hand, label-free techniques are beneficial in collecting cells without perturbing their functions. These techniques are also advantageous when the specific marker for the target cell is not fully known (Dharmasiri et al., 2010). Label-free manipulation is commonly achieved with one of two strategies: (1) direct sorting of target cells using the cells' intrinsic properties without labels (Durmus et al., 2015) and (2) indirect sorting of target cells by depleting unwanted cells in the surrounding medium (Iinuma et al., 2000; Lara et al., 2004; Bhuvanendran Nair Gourikutty et al., 2016a). Recently, microfluidic systems employing labeled or label-free sorting of rare cells have progressed to offer higher levels of control, purity, rapidness, and portability required for research and clinical applications (Table 1).

**Table 1:** Microfluidic rare cell separation applications based on positive and negative magnetophoresis.

**Cell type**	**Method**	**Surface marker**	**Magnetic element**	**Purity**	**Cell concentration**	**Capture rate**	**Throughput**	**Viability**	**References**
**STEM AND PROGENITOR CELLS**									
Endothelial progenitor cells (EPCs)	PM -MNP internalization (positive enrichment)	NU	NdFeB permanent magnet	NR	NR	40%	0.3 mL h^−1^	No difference between treated and untreated cells after 24 h in viability and tube formation function was reported	Kim et al., 2009
Hematopoietic stem cells (HSCs)	PM -MNP labeling (positive enrichment)	CD34	Permanent magnet	NR	5 × 10^7^ cells mL^−1^	88%	0.15 mL h^−1^	NR	Wu et al., 2010
Hematopoietic stem cells (HSCs) and endothelial progenitor cells (EPCs)	PM -MNP labeling(positive enrichment)	CD133	Electromagnetic wire	NR	HSC: ~6750 cells mL^−1^ EPC: ~1190 cells mL^−1^	HSC: > 96% EPC: > 95%	>14 mL h^−1^	No adverse effect on cell viability	Plouffe et al., 2012
Mouse lung multipotent stem cells (MLSCs)	PM -MNP labeling (negative and positive enrichment)	CD45 (-) CD31 (-) FITC (+)	Magnet	96–99%	NR	NR	1.2 mL h^−1^	Good self-renewal and proliferation capacity was obtained	Zeng et al., 2015
**TUMOR CELLS**									
Human colon adenocarcinoma cells (COLO205) and human breast cancer cells (SKBR3)	PM - MNP labeling (positive enrichment)	EpCAM	NdFeB permanent magnet	NR	5–1,000 cells mL^−1^ (TC: RBC = ~1: 10^7^- 10^9^)	COLO205: 90% SKBR3: 86%	10 mL h^−1^	NR	Hoshino et al., 2011
Mouse metastatic breast cancer cells (M6C)	PM - MNP labeling (positive enrichment)	EpCAM	NdFeB permanent magnet	<0.4% WBC capture	2–80 cells mL^−1^	~90%	1.2 mL h^−1^	> 90%	Kang et al., 2012
Breast cancer cells (MCF-7) and lung cancer cells (HCC827)	PM -MNP labeling (positive enrichment)	EpCAM	Permanent magnet	NR	~10 cells mL^−1^	~80%	NR	NR	Yoo et al., 2016
Human breast cancer cells (MCF-7 and MDA-MB-23)	PM -MNP labeling (positive enrichment)	EpCAM	Permanent magnet	NR	10^3^- 10^5^ cells mL^−1^ (MCF-7: MDA-MB-231 = 1:1)	MCF-7: 95.7% MDA-MB-23: 79.3%	3 mL h^−1^	NR	Kwak et al., 2017
Human acute monocytic leukemia cells (THP-1)	PM -MNP labeling (positive enrichment)	CD45	NdFeB permanent magnet	NR	10^6^ cells mL^−1^	NR	4.2 mL h^−1^	NR	Huang et al., 2018
Human colon cancer cells (COLO205)	PM -MNP labeling (positive enrichment)	EpCAM	Permanent magnet + nickel micromagnets	NR	~60 cells mL^−1^	Increased by 19% compared to no-micromagnet condition	2.5 mL h^−1^	NR	Chen et al., 2015
Breast cancer cells (SKBR-3)	PM -MNP labeling (positive enrichment)	EpCAM	NdFeB permanent magnet + ferromagnetic nickel-iron wires	97%	7 × 10^1^-6 × 10^4^ cells mL^−1^	90%	2–5 mL h^−1^	100% of the isolated cells were intact	Kim et al., 2013b
Breast cancer cells (MCF-7)	PM -MNP labeling (positive enrichment)	EpCAM	NdFeB permanent magnet + ferromagnetic nickel-cobalt wires	NR	1.25−2.5 × 10^5^ cells mL^−1^	93%	2.4–6 mL h^−1^	NR	Park et al., 2015
Breast cancer cells (MCF-7)	PM -MNP labeling (positive enrichment)	EpCAM	NdFeB permanent magnet + ferromagnetic nickel-iron wires	6.9–67.9%	1–10 cells mL^−1^	99.08%	4 mL h^−1^	NR	Cho et al., 2016
Human lung cancer cells (A549)	PM -MUNP labeling (positive enrichment)	EpCAM	Permanent magnet + silicon wires	NR	5 × 10^3^ cells mL^−1^	~90%	~1 mL h^−1^	Re-collected cells showed almost the same morphology compared to control cells	Wang et al., 2015
(i) Lung cancer cells (H-1650) (ii) Lung cancer cells (HCC827 and H-1650), breast cancer cells (MCF-7), human prostate cancer cells (LNCaP and PC-3) and human bladder cancer cells (T24) (EpCAM epxression levels: ~2000/cell to ~500,000/cell)	PM -MNP labeling (positive enrichment)	EpCAM	NdFeB permanent magnet + nickel-iron coated magnetic sifter	NR	(i) 4–470 cells mL^−1^ (ii) 50-100 cells mL^−1^ (>90% for >100 k EpCAM/cell)	H-1650: 95.7% HCC827, H-1650, MCF-7, LNCaP: 90% PC-3: 48%, T24: 17.7%	10 mL h^−1^	Unchanged cell viability was obtained	Earhart et al., 2014
Human ovarian cancer cells (HeLa)	PM -MNP labeling (positive enrichment)	EpCAM	Nickel-iron -based microstripline	NR	10^6^ cells mL^−1^	79%	0.06 mL h^−1^	100% cell viability was obtained with cooling	Wong et al., 2016
B lymphoid cells (Raji cell line) (target) T lymphoid cells (Jurkat cell line) (non-target)	PM -*in situ* magnetic labeling (positive enrichment)	CD19	Cooled electromagnet coil + microcontact printed ferrofluidic dots	96%	2 × 10^6^ cells mL^−1^ (Raji cells to total = 33%)	94%	3.6 × 10^4^ – 3.6 × 10^5^ total cells h^−1^	Viable cells with ability to move and divide were reported	Saliba et al., 2010
Human lung cancer cells (A549)	PM - *in situ* magnetic labeling (positive enrichment)	WGA modification	Magnetic solenoid coil + nickel micropillars	~93%	1.5 × 10^5^ cells mL^−1^	62–74%	NR	NR	Liu et al., 2007
Human T-lymphocytic leukemic cells (JM) and human ovarian cancer cell (HeLa)	PM -MNP labeling (positive enrichment)	CD4	NdFeB permanent magnet	>90%	~2 × 10^6^ mL^−1^	NR	~3.6 × 10^5^ cells h^−1^	NR	Mizuno et al., 2013
Breast cancer cells (MCF-7)	PM -MNP labeling (positive enrichment)	EpCAM	NdFeB permanent magnets +	NR	10^3^−3.3 × 10^4^ cells mL^−1^	up to 88%	~0.1 mL h^−1^	NR	Kirby et al., 2015
Lung carcinoma cells (H1299-GFP)	PM -MNP labeling (negative enrichment)	CD45	Permanent magnet	~50%	10^1^-10^5^ cells mL^−1^	~90%	60 mL h^−1^	>90%	Jiang et al., 2017
Human ovarian cancer cells (HeLa)	PM -MNP internalization (positive enrichment)	NU	NdFeB permanent magnet	NR	5 × 10^5^ cells mL^−1^	NR	NR	NR	Pamme and Wilhelm, 2006
Breast cancer cells (MDA-MB-231)	PM -magnetic susceptibility difference (negative enrichment)	NU	Permanent magnet +ferromagnetic nickel wire	NR	NR	94.8%	0.0025–0.0200 mL h^−1^	NR	Han et al., 2006
Breast cancer cells (SKBR3, MDA-MB-231) Prostate cancer cells (PC3-9)	PM -MNP labeling (positive enrichment)	EpCAM	Quadrupole magnetic circuit	>3.5-log purification resulted in 1,500 WBCs mL^−1^	200–1,000 cells mL^−1^	SKBR3: 98.6 ± 4.3% MDA-MB-231: 77.8 ± 7.8% PC3-9: 89.7 ± 4.5%	8 mL h^−1^ (3.6 × 10^10^ cells h^−1^)	Viable cells were obtained	Ozkumur et al., 2013
Human breast cancer cells (MCF10A and MCF10A-LBX1)	PM -MNP labeling (negative enrichment)	CD45 CD15		2.5-log purification resulted in 32,000 WBCs mL^−1^		MCF10A: 96.7 ± 1.9% MCF10A: 97.0 ± 1.7%			
Human melanoma cells (WM164), breast cancer cells (MB231, SKBR3), human lung cancer cells (PC9) and prostate cancer cells (PC3-9)	PM -MNP labeling (negative enrichment)	CD66b CD45	Permanent magnet	3.8-log purification	~10^3^ cells mL^−1^	97%	8 mL h^−1^ (3.6 × 10^10^ cells h^−1^)	NR	Karabacak et al., 2014
Human melanoma cells (SkMel28), lung cancer cells (H1650, H1975, H3122), prostate cancer cells (NCAP, PC3, PC3-9, VCAP) and breast cancer cells (MB231, MCF-7, SkBR)	PM -MNP labeling (negative enrichment)	CD66b CD45 CD16	Magnetic circuit	Purification resulted in 445 WBC mL^−1^	19–5,000 cells mL^−1^	99.5%	5.4−7.2 × 10^10^ cells h^−1^	NR	Fachin et al., 2017
Human colon cancer cells (HCT8)	PM -MNP labeling (negative enrichment)	CD45	Permanent magnet	Purification resulted in 83.99± 1.00% WBC depletion	10^4^ cells mL^−1^	70 ± 5% (for single round of depletion)	NR	Unchanged cell viability when a pulsation frequency of 0.05 Hz was used	Luo et al., 2015
Colorectal adenocarcinoma cells (HT29)	PM -MNP labeling (negative enrichment)	CD45	Magnet + layer of NdFeB magnetic grains	NR	50- 250 cells mL^−1^	87–96%	5 mL h^−1^	NR	Chung et al., 2013
Oncogenic human monocyte cells (U937)	NM	NU	NdFeB permanent magnet + nickel microstructure	>90%	8 × 10^7^ cells mL^−1^ (U937: RBC = 1:400)	NR	10^5^ cells h^−1^	NR	Shen et al., 2012
Breast cancer cells (MDA-MB-231), colorectal cancer cells (HCT116 and HT29), lung cancer cells (HCC827) and esophageal cancer cells (JHEsoAD1)	NM	NU	NdFeB permanent magnet	NR	NR	NR	No flow	Unchanged cell viability for long term cultivation in paramagnetic medium was reported	Durmus et al., 2015
Breast cancer cells (MDAMB-231), lung cancer cells (A549), ovarian cancer cells (HEYA8) and prostate cancer cells (PC-3)	NM	NU	NdFeB permanent magnet	NR	NR	NR	0.36 mL h^−1^	NR	Amin et al., 2017

*PM, positive magnetophoresis; NM, negative magnetophoresis*.

*NR, not reported; NU, not used*.

*Capture rate, the ratio of the number of cells collected after separation to the total number of cells loaded to the chip*.

*Purity, the ratio of the number of target cells collected after separation to the total number of collected cells*.

#### Stem Cells

**Figure 2:**
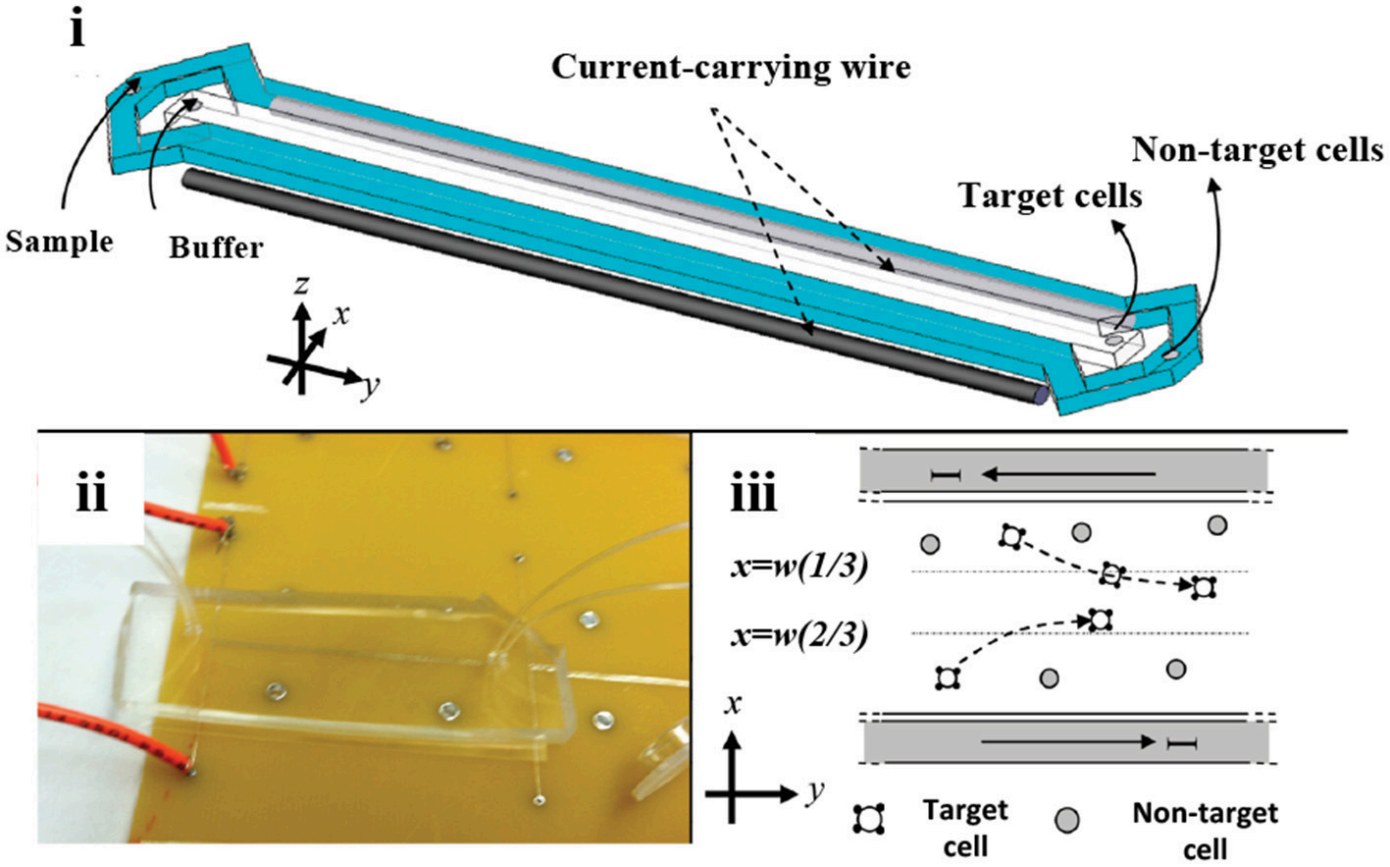
Microfluidic stem cell separation device. Magnetophoretic HSC and EPC separation based on anti-CD133-conjugated magnetic labeling. **(i)** Illustration and **(ii)** photograph of the device. **(iii)** Magnetic field created by electromagnetic wires deflects magnetically-labeled stem cells into a center collection stream. Reprinted with permission from Plouffe et al. (2012). Copyright (2012) American Chemical Society.

Innovative designs using microfluidic principles for separation of SCs have been mostly tested with immunomagnetic labels. Kim et al. designed a microfluidic system to model a blood vessel, aiming to solve losses in injected number of SCs for therapeutic *in vivo* applications (Kim et al., 2009). Localization of endothelial progenitor cells (EPCs) to a specific site within a polydimethylsiloxane (PDMS)-based microfluidic channel was simulated. The method included the incorporation of *Magnetospirillum* sp. AMB-1-based magnetic nanoparticles (10 μg MNP /10^4^ cells) into EPCs. About 40% of magnetically-tagged EPCs flowing in the channel adhered to the microfluidic channel wall under an external magnetic field. Most of the EPCs maintained their viability and tube formation function. Later, Wu et al. developed a microfluidic platform consisting of isolation, counting, and sorting modules on a single chip for isolation of hematopoietic stem cells (HSCs) from cord blood (Wu et al., 2010). Plouffe et al. also studied the isolation of HSCs and EPCs from whole blood in a PDMS-based chip containing an electromagnetic wire array (Plouffe et al., 2012) (Figure 2). First, HSCs and EPCs were pre-labeled and isolated using anti-CD133 functionalized MNPs from whole blood. For further identification of isolated HSCs and EPCs, cells were labeled with antibodies against CD34, CD45 and kinase insert domain receptor (KDR). A magnetic field was created by two wires on the top and bottom of the microfluidic separation chamber. Under the magnetic field, labeled HSCs (CD133+/CD34+/CD45+/KDR-) and EPCs (CD133+/CD34+/CD45-/KDR+) were deflected from the two lateral sample streams to a central buffer stream according to the magnetic properties of individual cells. Separation efficiency was determined as >96%. Another immunomagnetic microfluidic sorting was performed for the isolation of mouse lung multipotent stem cells (MLSCs) (Zeng et al., 2015). First, magnetically-labeled cells (i.e., CD45+ and CD31+) were directed into the isolation zone of the microfluidic platform (IsoFlux^TM^) using an external magnet, whereas unlabeled cells flowed through the unlabeled cell zone. Second, the unlabeled cells were collected and labeled with FITC-coupled Sca-1 (Stem cell antigen-1) antibody. Then, using anti-FITC magnetic beads, MLSCs (CD45-, CD31-, and Sca-1+) were collected at the isolation zone. The efficiency was determined as 96–99% by FITC signal and isolated MLSCs retained their capacity for self-renewal and differentiation.

#### Circulating Tumor Cells

Positive magnetophoresis based on labeling cells with MNPs have been applied frequently in the last decade for CTC sorting. Hoshino et al. developed a PDMS-based microchip using anti-EpCAM antibody-functionalized MNP (Veridex Ferrofluid^TM^) to separate COLO205 and SKBR3 cancer cells spiked in the blood (Hoshino et al., 2011). The chip captured MNP-conjugated cancer cells by an array of three permanent magnets with alternating polarities. Average capture rates were 90% and 86% for COLO205 and SKBR3 cells, respectively. Kang et al. also used anti EpCAM-functionalized magnetic beads (2.8 μm) to separate EpCAM + breast cancer cells (BCCs) from blood in a microfluidic chip (Kang et al., 2012). The design consisted of an inlet, the main channel and double collection channels before the outlet. The inlet channel contained a micropillar array (50 × 50 μm) that filter the sample fluid from cell and bead clusters to prevent clogging. Main and double collection channels were lined by rows of dead-end collection side chambers. Magnetically-labeled BCCs were pulled from the flow through the collection side chambers while white blood cells (WBCs) moved through the channel with the sample flow under an external magnetic field. The BCCs were separated with high efficiency (87%), specificity (< 0.4% WBC capture) of WBC captured, and viability (>90%). Later, Yoo et al. used anti-EpCAM magnetic particles in a system containing separation and collection wells for vertical magnetic isolation of spiked CTCs (MCF-7 and HCC827) in plasma-depleted blood (Yoo et al., 2016). Recently, Kwak et al. used anti-EpCAM MNPs to isolate human BCCs with different EpCAM expression levels [i.e., MCF-7 (EpCAM +) and MDA-MB-231 (EpCAM -)] in the microchannels with five serpentine-trapping segments (Kwak et al., 2017). Trapping segments with 30 rectangular subsegments were perpendicular to the flow. A magnetic field gradient was created perpendicular to the flow at the end of the trapping segments. CTCs flowing in the chip were collected at different segments depending on the extent of their magnetic label. For instance, high EPCAM-expressing MCF-7 bore higher anti-EpCAM magnetic particles and they were attracted by the magnetic force and collected mostly at the segments located most distantly from the magnet. The chip separated CTCs with an average yield of 97.5% and 79.3% for EpCAM+ and EpCAM- CTCs, respectively. Recently, anti-CD45-based immunomagnetic labels were used in a microfluidic device containing an attached microwell layer between a microchannel and a permanent magnet to collect individual THP-1 cells in the microwells (Huang et al., 2018).

**Figure 3:**
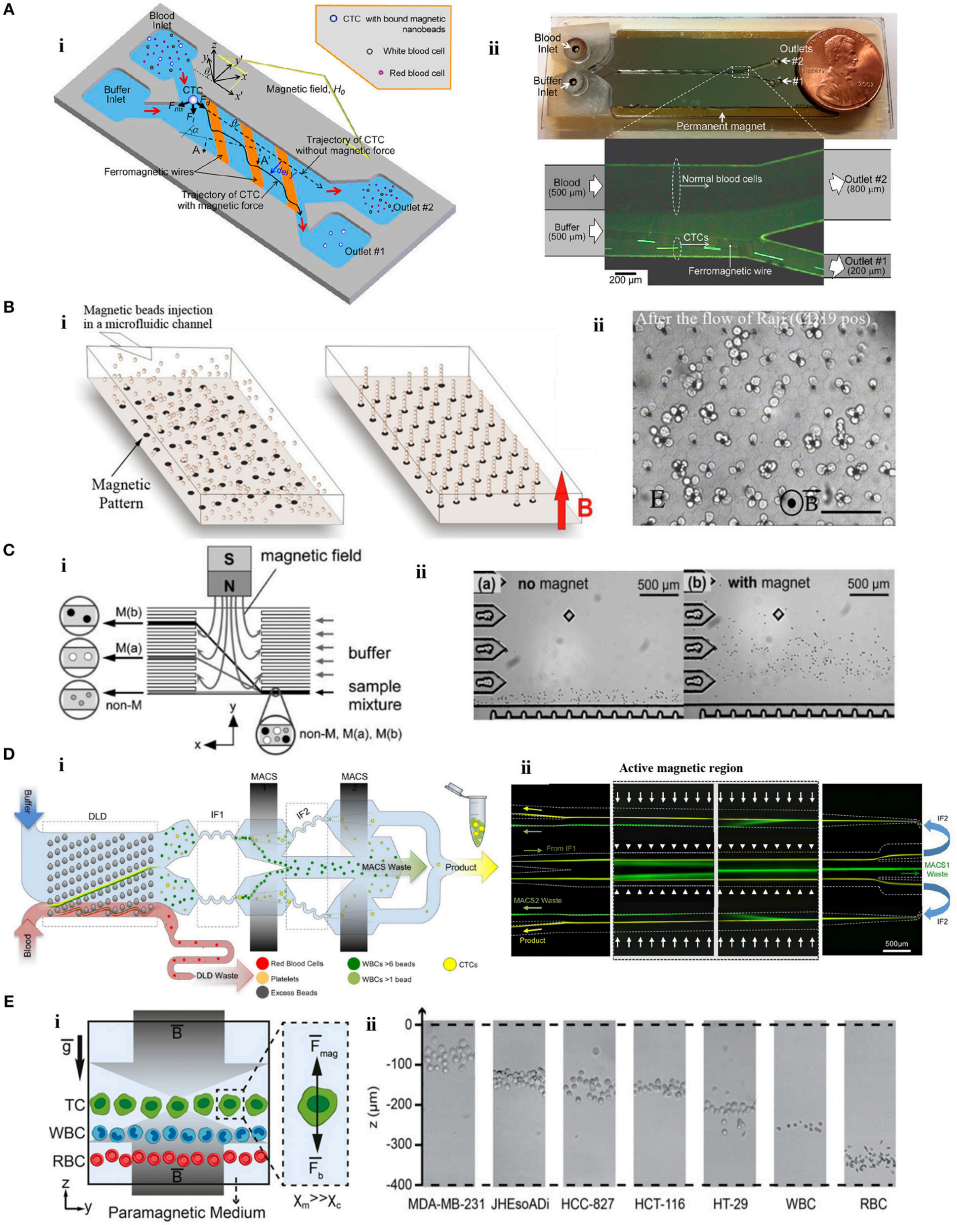
Microfluidic tumor cell separation devices. **(A)** Separation of magnetically-labeled target cells. **(i)** Design and **(ii)** optical micrograph of the positive tumor cell (SKBR-3) enrichment device using anti-EpCAM-coupled magnetic nanoparticles. Reprinted with permission from Kim et al. (2013b). Copyright (2013) American Chemical Society. **(B)** *In situ* magnetic labeling of tumor cells (Ephesia). **(i)** The magnetic beads are located on ferrofluid dots in the microfluidic channel to create a self-assembled magnetic bead array under the applied magnetic field. **(ii)** Cells are captured on the magnetic bead array during the sample flow. Reprinted from Saliba et al. (2010). **(C)** Separation of tumor cells (HeLa) based on MNP uptake extent. **(i)** The schematic illustration of the device. **(ii)** HeLa cells are deflected from the laminar flow according to their magnetic load. Reprinted with permission from Pamme and Wilhelm (2006). Copyright (2006) The Royal Society of Chemistry. **(D)** Separation of cancer cells by depleting other cells. **(i)** A monolithic microfluidic chip for negative enrichment of tumor cells through the depletion of magnetically-labeled WBCs. In the deterministic lateral displacement (DLD) part, RBCs, platelets, and free beads are eliminated. Following the inertial focusing 1 (IF1), magnetophoresis (MACS1) is applied to deplete magnetic labeled WBCs having more than ~6 beads. Another set of inertial focusing (IF2) and magnetophoresis (MACS2) is applied to deplete WBCs that contain at least one magnetic bead. **(ii)** Image of cell streaks captured using fluorescence microscopy on different areas of the chip. Green and yellow colors represent WBCs and CTCs, respectively. Reprinted from Fachin et al. (2017). **(E)** Tumor cell separation using cells' intrinsic properties in a paramagnetic solution. **(i)** The design of the label-free magnetophoresis platform (MagLev). **(ii)** Alignment of tumor cells at different heights (*z-axis*) in the device. Reprinted from Durmus et al. (2015).

Modulating a magnetic field using microscale magnetic elements is important in various applications (Lee et al., 2001, 2004; Kimura et al., 2004), including cell sorting. An example of these devices was a 2D micromagnet array for CTCs (Chen et al., 2015). In the device, patterned thin-film micromagnets were used together with an external magnet to enhance the effect of the external magnetic field locally and create discontinuous capture sites for CTCs. The array was tested with magnetically-tagged human colon cancer cells (COLO205) spiked in blood samples. Throughout the assay, blood cells moved with the flow in the microfluidic chamber while anti-EpCAM magnetic label-CTC complexes were captured alongside the array. The results revealed that pattern integration increased the average capture rate and distribution uniformity by 19% and 14%, respectively, compared to the device without micromagnets. Another approach was developed including the use of inlaid ferromagnetic wires and an external permanent magnet in a microseparator device (Kim et al., 2013b). Anti-EpCAM labeled CTCs (SKBR-3) were separated from blood cells, and directed to a separate outlet in the device (Figure 3A). Spiked CTCs were isolated with a purity of 97% and a yield of 90%. The device was also tested for the peripheral blood of breast and lung cancer patients. Based on a principle similar to Kim et al., Park et al. used ferromagnetic Ni-Co nanowires in a microdevice for MCF-7 cells. Under an external magnetic field, anti-EpCAM MNP-labeled MCF-7 cells were collected from whole blood with a purity of 93% (Park et al., 2015). Cho et al. also used ferromagnetic wires together with two permanent magnets for separation of anti-EpCAM magnetic bead-conjugated MCF-7 cells from red blood cell (RBC) -lysed whole blood (CTC-μChip) (Cho et al., 2016). The average recovery rate was 99.08%, and the purity of CTCs was in the range of 6.9–67.9% depending on the spiked CTC concentration. Another design included a magnetic sifter-based microstructure having arrays of 40 × 40 μm pores in a honey comb format to separate cancer cells (Earhart et al., 2014). MNP-anti-EpCAM-labeled H-1650 lung tumor cells spiked in whole blood were pushed with a fluid flow toward the patterned pores of the sifter. Magnetically-labeled cancer cells were captured on the magnetized sifter while other cells passed through the pores. The device separated tumor cells with a 91.4% efficiency. Later, the device was tested for cells (HCC827, H-1650, MCF-7, LNCaP, PC-3, T24) spiked in whole blood with varying EpCAM expression levels (~2000/cell to ~500,000/cell). For the cells with high EpCAM expression levels (HCC827, H-1650, MCF-7, LNCaP), the yield of the separation was as high as 90%; however, low EpCAM expressing PC-3 and T24 cells were captured with efficiencies of 48% and 17.7%, respectively. The device was also tested for enumeration of CTCs in the blood of non-small cell lung cancer patients as preliminary attempts. Microstrip lines, which are typically current conducting elements fixed on the device substrate, can produce a high magnetic field gradient in a tunable manner instead of simple off-chip permanent magnets. It was shown that deflection of magnetically-tagged (COMPEL™ 8 μm) human ovarian cancer (HeLa) cells by microstrip lines could achieve a separation with an efficiency of 79% (Wong et al., 2016). Lately, magnetic upconversion nanoparticles (MUNPs) have received much attention as novel probes for sensitive detection of biomolecules due to lack of autofluorescence. Wang et al. integrated anti-EPCAM-conjugated MUNPs in a microfluidic device with silicon-nanowire-arrays and an external magnet to capture a small number of cancer cells in spiked blood samples and also in clinical samples from lung cancer patients (Wang et al., 2015).

In addition to the batch-wise mixing of magnetic beads with cells, creating magnetic self-assembly bead arrays in a microfluidic chip using monoclonal antibody (mAb)- functionalized magnetic beads was reported for sorting of cancer cells (Ephesia) (Saliba et al., 2010) (Figure 3B). In the chip, beads were organized onto a microcontact printed ferrofluid dot pattern under a magnetic field. On the pattern, the dipole-dipole interaction between the beads created 3D-array of magnetic beads oriented in the direction of the field. The chip with the array of magnetic beads bearing anti CD-19 mAb was tested for a mixture of CD19- Jurkat and CD19+ Raji cells. Raji cells were captured by the array of beads with a yield of 94% while Jurkat cells moved with the flow. Another microfluidic device without off-chip labeling was developed to isolate A549 cancer cells from RBCs (Liu et al., 2007). First, superparamagnetic particles (260 nm) were trapped at the zone of a hexagonal array of Ni micropillars generating a strong magnetic field gradient under a controlled external magnetic field. Then, magnetic particles were functionalized *in situ* with corresponding antibodies, and A549 cancer cells were trapped and enriched by 133-fold (A549 to RBC ratio = 1:10).

Integrated microfluidic systems combining forces of differing natures have also been tested for CTCs. Mizuno et al. developed a 2D sorting device combining hydrodynamic filtration for size-based sorting and magnetophoresis for surface marker-based (CD4+) sorting of cancer cells (Mizuno et al., 2013). Kirby et al. merged magnetophoretic and centrifugal forces on a disposable PDMS cartridge as a lab-on-a-disc format for the separation of EpCAM+ MCF-7 cancer cells from blood (Kirby et al., 2015). Magnetically-tagged MCF-7 cells and blood were placed into the disc and rotated at a rate of 17 Hz. Under centrifugal and magnetic forces, 80% of tagged cancer cells were routed to a capture chamber. Lately, Jiang et al. combined deterministic lateral displacement (DLD) and immunomagnetic separation in a microfluidic device (Jiang et al., 2017). The CTCs (H1299-GFP) spiked in blood samples were captured with an efficiency and purity of ~90 and 50%, respectively. The device was also tested with the blood of cancer patients and revealed promising results for potential clinical applications.

Besides manipulation depending on surface marker expression, magnetic particle uptake extent was utilized to distinguish cancer cells using a microfluidic chip (Pamme and Wilhelm, 2006) (Figure 3C). The chip contained a separation chamber in which a laminar flow was provided perpendicular to the direction of the magnetic field. Magnetic nanoparticle-loaded [i.e., maghemite nanoparticles (γFe_2_O_3_)] HeLa cells that differ in their uptake capacity were deflected under an external magnetic field (~400 mT) from the laminar flow toward the different levels of the separation chamber according to their magnetic moment and size.

Another application of positive magnetophoresis is the enrichment of CTCs through depletion of unwanted cells by either labeling them (e.g., WBCs) with magnetic particles or using their intrinsic properties (e.g., RBC). An example of the latter was as a hybrid microsystem which included a paramagnetic capture module (PCM) and micro-electrical impedance spectroscopy (μ-EIS) (Han et al., 2006). The microsystem was tested for three BCCs (MCF-7, MDA-MB-231, and MDA-MB-435). In PCM (200 mT), deoxyhemoglobin RBCs with inherent paramagnetic properties were attracted by a ferromagnetic wire and depleted from the solution. Then in μ-EIS module, the impedance analysis of cancer cells was performed at the single cell level. Results revealed that 94.8% of the BCCs were separated and characterized in a continuous manner. Enrichment of tumor cells based on depletion of magnetically-labeled CD45+ lymphocytes was tested in an integrated device with micropumps and mixers (Luo et al., 2015). CD45- cancer cells (HCT8) were recovered with a 70% efficiency for a single round of enrichment. Similarly, in a magnetic/size-sorting device, a layer of magnetic grains depleted magnetically-labeled CD45+ leukocytes and a size-sorter captured and collected individual cancer cells (HT29) at predefined locations (Chung et al., 2013). In a device offering both positive and negative enrichment modes (Ozkumur et al., 2013; Karabacak et al., 2014), leukocytes labeled with magnetic beads were depleted from a blood sample in the negative enrichment mode (^neg^CTC-iChip). The ^neg^CTC-iChip platform consisted of two serial modules. In the first module, nucleated cells (WBCs and tumor cells) were separated from RBCs, platelets, and unbound beads by their sizes using DLD. In the second module, cells were lined-up by inertial focusing for better manipulation. Then, permanent magnets in a quadrupole orientation depleted WBCs. The chip isolated cancer cells with a 97% efficiency (Karabacak et al., 2014). Afterwards, the CTC-iChip was improved for better throughput and purity (Fachin et al., 2017) (Figure 3D). In this case, WBCs were depleted using two stages of magnetophoresis. In the first magnetic region (200 T/m), WBCs labeled with more than ~6 beads were depleted from the sample. Following a second set of inertial focusing, WBCs containing at least 1 magnetic bead were depleted in the second region (425 T/m).

In contrast to approaches with labels, negative magnetophoresis exploiting intrinsic physical biomarkers of cells, and therefore independent of magnetic tags or labels, is a simple and affordable technique. U937 cells were separated from RBCs by tuning resolution capacity of magnetophoresis in gadolinium (Gd) diethylenetriamine penta-acetic acid (DTPA)-based paramagnetic solutions (0–80 mM) (Shen et al., 2012). Under an external magnetic field applied perpendicular to the direction of flow and a Ni microstructure, cells were deflected laterally due to the magnetic repulsion force. The most efficient separation was achieved at 40 mM Gd–DTPA with a >90% purity. Gd-based paramagnetic solutions (0–100 mM) was tested for density-based, label-free, and real-time imaging of cells in a non-flow magnetic levitation platform as well (MagDense) (Durmus et al., 2015) (Figure 3E). Tumor and blood cells were levitated in a capillary between two opposing magnets (1.45 T). The characteristic levitation height depended on the intrinsic signatures of the cells: magnetic susceptibility and density. All types of tumor cells (i.e., HT29, HCT116, HCC827, JHesoAD1, and MDA-MB-231) spiked in Gd-based medium were levitated at distinguishably higher positions than blood cells. In addition, heterogeneity among seemingly homogeneous tumor cells populations was demonstrated at the single cell level. Recently, this density-based magnetic focusing has been applied in a platform made of a 3D-printed smartphone module and built-in camera for real time monitoring and spatial separation of cancer and blood cells (Amin et al., 2017; Knowlton et al., 2017).

### 2D and 3D Cell Culture

**Figure 4:**
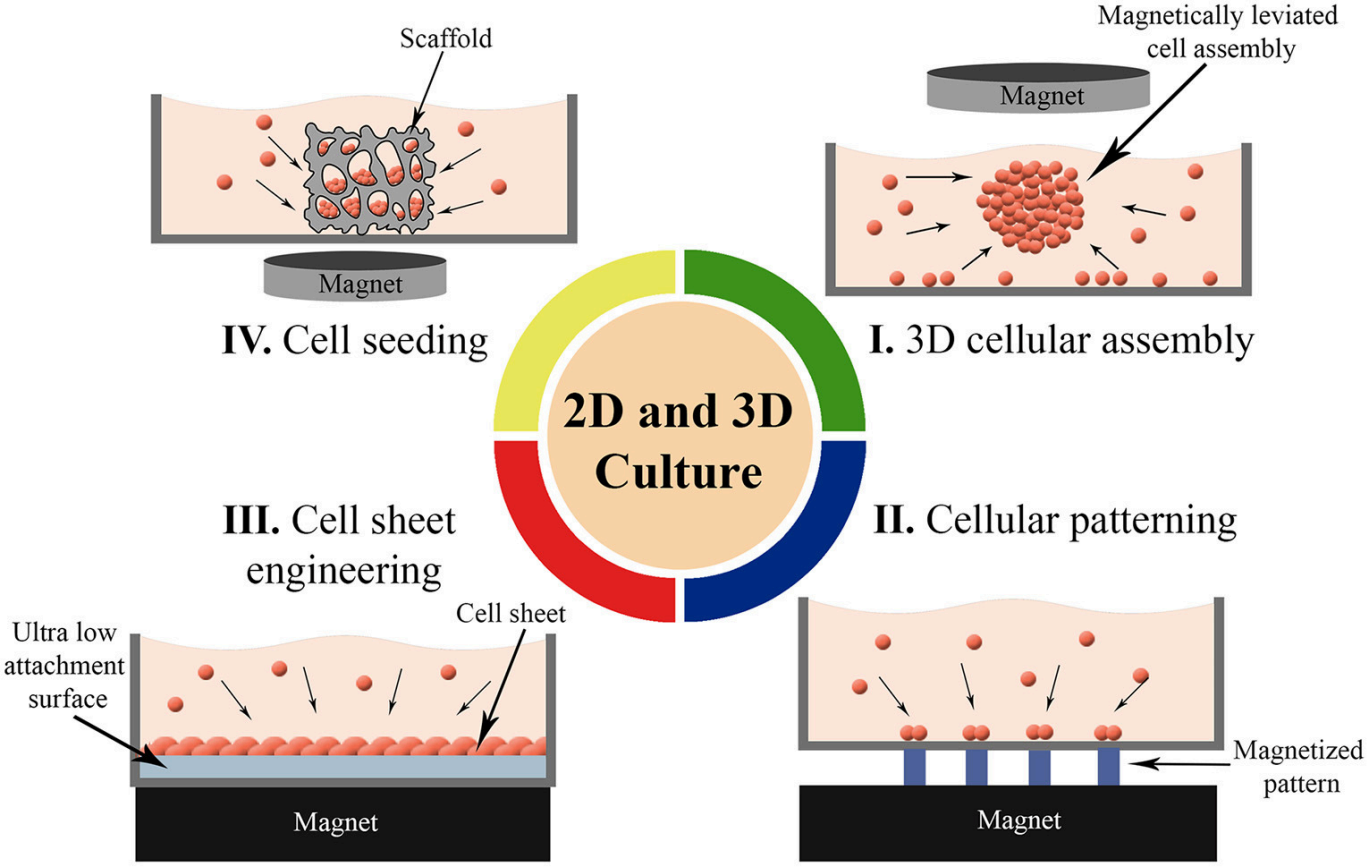
Magnetic force-based 2D and 3D cell culture techniques. **(I)** Formation of 3D cellular assembly as building blocks, **(II)** Organizing cells or spheroids into a targeted pattern, **(III)** Guiding cells into sheet-like structures for a close cellular contact, and **(IV)** Enhancing the seeding efficiency of the cells into scaffolds.

To meet functional requirements, organs arrange one or more cell types in specific forms (Rivron et al., 2009). Manipulation of cells in 2D and 3D cell culture that aims biomimicry is therefore crucial to reflect appropriate form and function. Thus, development of techniques to organize cells in targeted arrangements in convenient microenvironments is one of the most important trends in cell culture technologies. Magnetic cell manipulation techniques have been used for various purposes in 2D and 3D cell culture (Figure 4) such as; to form 3D cellular assembly (Table 2) as building blocks (Mattix et al., 2014a), to organize cells or spheroids into a targeted pattern (Ino et al., 2009; Whatley et al., 2014), to create cell sheets for a tight and close cellular contact (Ishii et al., 2014) and to increase cell seeding efficiency into scaffolds (Thevenot et al., 2008). Here, we reviewed recent advances in magnetic-based 2D and 3D cell culturing techniques that were either used in microfluidic devices or represent potency for future microfluidic applications.

**Table 2:** Summary of 3D cellular assembly applications.

**Manipulation strategy**	**Cell type**	**Biofabrication output**	**Microfluidic**	**References**
PM (labeling cells via internalization using bovine serum albumin coated MNP)	Human prostate cancer epithelial (PC-3) cells and human lung fibroblast (HFL-1) cells	Multilayer sheet structures for epithelial cells, tightly packed spheroids for fibroblasts after 24 h of manipulation.	-	Ghosh et al., 2016
PM (labeling cells via a hydrogel consisting of gold, iron oxide MNP and filamentous bacteriophage)	Normal human astrocytes and human glioblastoma (LN-229 or U-251MG)	Spheroids with ~ 930 μm diameter after 10.5 days of levitation	-	Souza et al., 2010
PM (labeling cells via NANOSHUTTLE™)	Preadipocyte cells (3T3-L1) and endothelial cells (bEND.3)	Adiposphere-based coculture with a vascular-like network assembly and lipogenesis in perivascular cells.	-	Daquinag et al., 2012
PM (labeling cells via NANOSHUTTLE™)	Primary human epithelial cells, smooth muscle cells, pulmonary fibroblasts, and pulmonary endothelial cells	3D bronchiole coculture consisting of four cell types together in a layered assembly after 7 days of levitation culture	-	Tseng et al., 2013
PM (labeling cells via NANOSHUTTLE™)	Primary porcine valvular interstitial cells and endothelial cells	3D layered co-culture model of the aortic valve with ~ 2800 μm diameter after 3 days of levitation	-	Tseng et al., 2014
PM (labeling cells via NANOSHUTTLE™)	Breast cancer cells (SUM159, MDA-MB-231) and fibroblasts (293T, Hs578bst, human pulmonary fibroblasts and patient derived tumor associated fibroblasts)	Large-sized (millimeter in diameter) co-culture model of breast tumor within 24 h	-	Jaganathan et al., 2014
PM (labeling cells via MNP)	Bone marrow-derived human MSCs	Random mixed, core-shell, and fused spheroids composed of cells stained with two different dyes with 100–200 μm in diameter	-	Kim et al., 2013a
PM (labeling cells via NANOSHUTTLE™)	Rat vascular smooth muscle cells (A10) and primary human aortic smooth muscle cells	Contractile rings with ~ 3 mm in outer diameter	-	Tseng et al., 2016
PM (labeling cells via magnetite cationic liposomes)	Mouse myoblast cells (C2C12)	Cell sheets with 0.63 cm^2^ area after 24 h, cell strings with ~150 μm in longitudinal direction after 24 h, cell rings with 12 mm in diameter after 48 h	-	Yamamoto et al., 2009, 2010
PM (labeling cells via magnetite cationic liposomes)	Primary neonatal rat cardiomyocytes	Cardiac tissue rings with ~250 μm thickness after 7-day cultivation	-	Akiyama et al., 2010
PM (labeling 3D cellular spheroids via incorporation of magnetoferritin nanoparticles into spheroids)	Primary rat aortic smooth muscle cells	Tissue rings formed by fusion of spheroids over 4 days (~ 13 mm in diameter)	-	Mattix et al., 2014a
PM (labeling 3D cellular spheroids via incorporation of MNP into ECM of spheroids)	Primary rat aortic smooth muscle cells	Tissue rings (from 2 mm up to 10 mm) and custom patterns (square and Clemson University Tiger Paw) formed by fusion of magnetic labeled spheroids over 4 days	-	Mattix et al., 2014b
PM (labeling cells via deposition of poly(allylamine)-stabilized MNP on cell membranes)	Primary human skin fibroblasts (HSF) and human lung carcinoma epithelial cells (A549)	Layered planar tissue constructs (~100 μm thick, round, and 3 mm in diameter) after 24 h incubation of surface-engineered magnetic cells	-	Dzamukova et al., 2015
PM (forming magnetic 3D cellular structures via adhesion of cells to magnetic iron oxide-encapsulated nano/microparticle substrates)	Human epidermoid tumor KB cells	Tumor cell spheroids with an increase in volume during 10-day culture period	-	Lee et al., 2011
PM (forming magnetic 3D cellular structures via adhesion of cells to magnetic collagen hydrogel beads)	Mouse fibroblast cells (NIH-3T3) and human hepatocellular carcinoma cells (Hep G2)	Magnetically manipulable cells adhered on the collagen beads	-	Sugaya et al., 2012
PM (labeling biotinylated cells via streptavidin paramagnetic particles)	Human embryonic kidney cells (HEK293) and human breast cancer cells (MCF-7)	Magnetically orientable cells and spheroids in hanging drop culture to target and immobilize spheroids for a facilitated media change and therapeutic screening, covering different cells onto preformed spheroids	-	Ho et al., 2013
NM (suspension of cells in paramagnetic solution containing gadolinium diethylenetriaminepentaacetic acid)	Bovine carotid artery cells (HH)	Egg-shaped cellular structure with 510 μm diameter and 690 μm height in 20 min	-	Akiyama and Morishima, 2011a
NM (suspension of cells in paramagnetic solution containing gadoteric acid)	Bovine carotid artery cells (HH)	Spheroids with ~400 μm in diameter after one day of culture (25 spheroids in each batch)	-	Akiyama and Morishima, 2011b
NM (suspension of cells in paramagnetic solution containing gadoteric acid)	Mouse myoblast cells (C2C12)	Spheroids with ~250 μm diameter within 1 min	+	Akiyama and Morishima, 2012
NM (suspension of cells in paramagnetic solution containing gadolinium diethylenetriaminepentaacetic acid)	Whole blood cells	Rectangular bar, three-pointed star shaped cellular structures and spheroids of varying sizes (600–1,000 μm)	-	Abdel Fattah et al., 2016
NM (suspension of cells in paramagnetic solution containing Gadavist®)	Murine fibroblasts (NIH 3T3)	Cellular clusters (100–260 μm) formed by magnetic levitation after 48 h, merged preformed-spheroids after 4 days and assembly of cells compartmentalized in the water-in-oil droplets after 24 h	+	Tocchio et al., 2017
NM (suspension of spheroids in paramagnetic solution containing Omniscan™)	Primary sheep chondrocytes	Fused chondrospheres	-	Parfenov et al., 2018
NM (suspension of cells in paramagnetic solution containing Gadavist®)	Bone marrow stem cells (D1 ORL UVA) and breast cancer cells (MDA-MB-231)	Cellular blocks up to ~2.7 cm in length (with ~280 μm thickness) formed by magnetic levitation after 48 h and biphasic cellular structures in a single device	+	Anil-Inevi et al., 2018
NM (suspension of cells in paramagnetic solution containing Gadavist®)	Mouse fibroblast cells (NIH 3T3) and non-small-cell lung cancer cells (HCC827)	Cell spheroids and cell strings with increase in cell number during 168-h culture	+	Türker et al., 2018
PM (forming magnetic 3D cellular structures via encapsulation of cell within paramagnetic hydrogel)	Mouse fibroblast cells (NIH 3T3)	Magnetically controllable cell-encapsulating hydrogels with manufacturability in different sizes (150 μm in thickness and 200–1,000 μm in side dimension)	-	Tasoglu et al., 2013
NM (suspension of cells in paramagnetic solution containing gadolinium diethylenetriaminepentaacetic acid)	Mouse fibroblast cells (NIH 3T3)	Assembled building blocks; cell encapsulating hydrogels (2 mm round with 150 μm thickness) and cell seeded microbeads	-	Tasoglu et al., 2015b
PM (manipulation of cell encapsulating hydrogels via motion of the magnetic microrobots)	Human umbilical vein endothelial cells (HUVECs), mouse fibroblast cells (NIH 3T3), cardiomyocyte	2D and 3D heterogeneous assembly of cell encapsulating hydrogels.	-	Tasoglu et al., 2014

*PM, positive magnetophoresis; NM, negative magnetophoresis; MNP, magnetic nanoparticles; NANOSHUTTLE™, assembly of iron oxide and gold nanoparticles cross-linked with poly-l-lysine*.

*–, The shortest dimension of the cell culture chamber > 1 mm*.

*+, The shortest dimension of the cell culture chamber ≤ 1 mm*.

#### 3D Cellular Assembly

**Figure 5:**
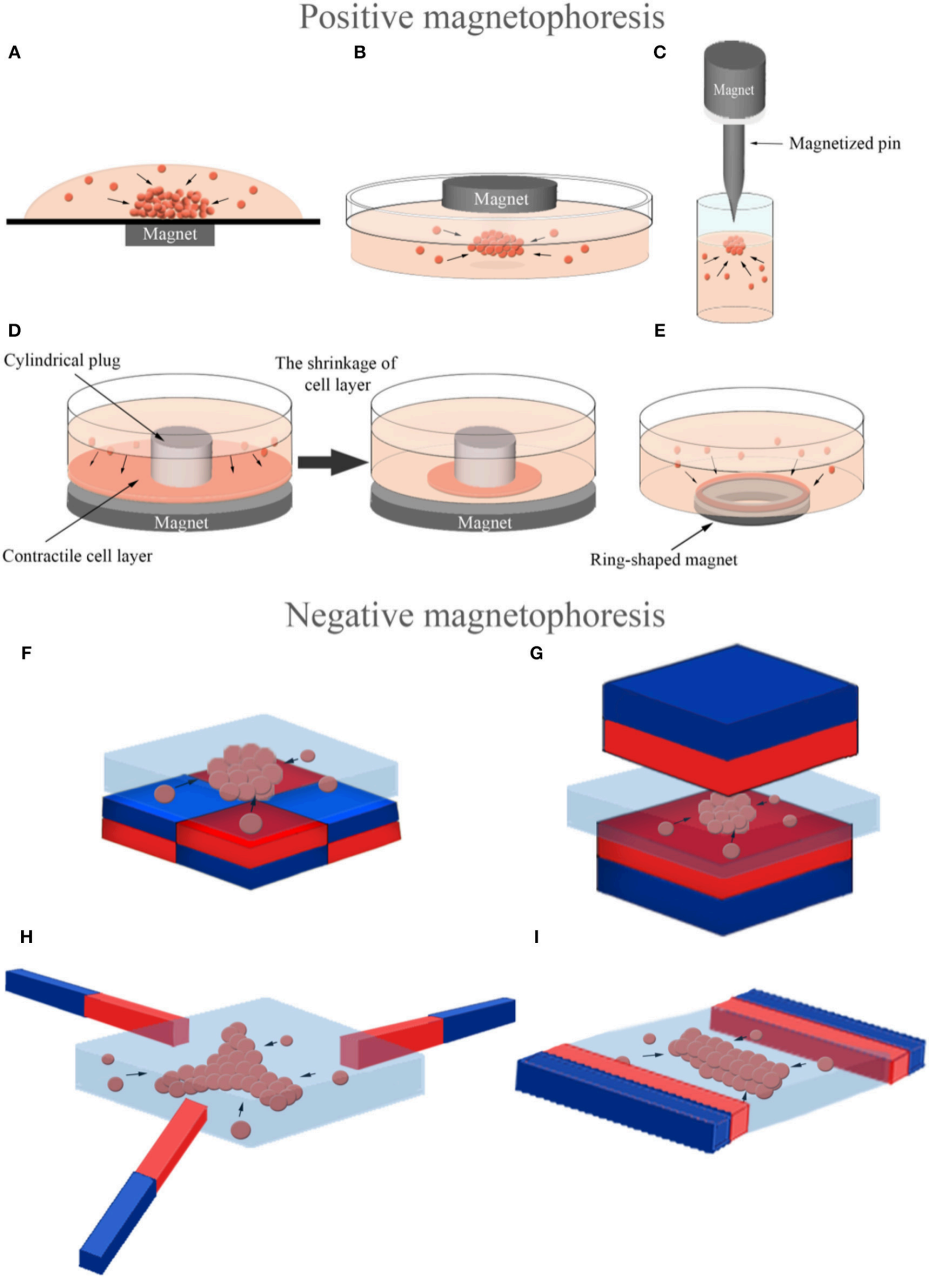
Magnetic force-based manipulation of magnetically labeled cells (positive magnetophoresis) and label-free diamagnetic cells (negative magnetophoresis). 3D assembly of magnetically labeled cells into a spheroid by a magnet **(A)** under the culture chamber, **(B)** on the top of the culture chamber and **(C)** by a magnetized pin beneath the magnet to concentrate the magnetic field for attracting cells in a focused direction. 3D assembly of magnetically labeled cells into a ring-shaped structure **(D)** using a cylindrical plug and a magnet under it to accumulate contractile cells around the plug and **(E)** using a ring-shaped magnet. 3D assembly of label-free diamagnetic cells into **(F,G)** spheroid, **(H)** three-pointed star and **(I)** rectangular bar in a magnetic liquid with different configurations of magnets to produce a spatially varying field along the culture chamber (The north poles: red, the south poles: blue).

3D cellular spheroid culture is a valuable tool to mimic the tissue-specific properties of cells (Lin and Chang, 2008). Magnetic force-based guidance of cells into spheroids offers a unique way to form 3D arrangements in a non-contact mode. Cells can be aggregated into spheroids using positive magnetophoresis by assembly of cells at a certain location on the surface of a culture chamber above permanent magnets with a multi-step seeding process (Figure 5A) where cluster shape is determined by cell type used (Ghosh et al., 2016). Positive magnetophoresis can also assemble cells at the air–medium interface (Figure 5B) (Souza et al., 2010; Jaganathan et al., 2014) with magnetic levitation. Souza et al. reported this levitation-based 3D cell culture method using a bioinorganic hydrogel comprised of filamentous bacteriophage, MNPs and gold nanoparticles (Souza et al., 2010). Hydrogel-treated cells were levitated to the air–medium interface with a permanent magnet and manipulated for the formation of different 3D geometries and cellular compositions by spatial variations in the magnetic field. A second-generation, bacteriophage-free hydrogel was also described to enable the magnetization of cells without the use of any toxic or infectious agents (Souza, 2013) and commercialized under the trade name NANOSHUTTLE™ (NS) and the Bio-Assembler™ kit that contains the NS solution and a magnetic drive.

There are several examples that use magnetic cell guidance to form tissue-specific constructs. Daquinag et al. developed a 3D tissue culture system based on cell levitation via MNP-labeling to model white adipose tissue development (Daquinag et al., 2012). 3T3-L1 preadipocytes remained viable in levitated spheroids for extended periods, while 2D cultured cells died after reaching confluence. Adipogenic-induced adipospheres composed of 3T3-L1 cells successfully mimicked *in vivo* white adipocytes by formation of large lipid droplets unlike 2D cultured ones. Magnetic levitation with MNPs was also used to assemble a 3D co-culture structure of the bronchiole with sequential layers of epithelial cells, smooth muscle cells, pulmonary fibroblasts, and pulmonary endothelial cells in a similar organization to native tissue (Tseng et al., 2013). The resulting co-culture maintained the phenotype and induced extracellular matrix (ECM) formation in contrast to 2D culture. Same levitation-based co-culture model was also successfully utilized in creating sequentially assembled 3D structures of aortic valve cells; valvular interstitial and endothelial cells (Tseng et al., 2014). The resulting 3D model maintained cell phenotype and function, and synthesized relevant ECM molecules. Additionally, a novel *in vitro* model mimicking heterogeneous breast tumors was developed by 3D cellular assembly of BCCs and fibroblasts by magnetic levitation, which can control tumor composition for microenvironment in an effort to test anti-cancer drugs (Jaganathan et al., 2014). A magnetic pin-array system was also developed to assemble MNP-incorporated cells into 3D spheroids (Kim et al., 2013a). In this system, an iron pin and magnet were combined to generate a concentrated and strong magnetic field at a specific point and thus to reinforce cell-cell contact in 3D spheroids (Figure 5C). The method provided accurate control over spheroid size and had versatile applications as it was able to create random mixed, core-shell, and fused. In addition to 3D spheroids, another group of studies reported guiding magnetically-labeled cells were guided into ring-shaped structures at the macro level. In these studies, ring-shaped muscle tissues were created either by a system that allowed the cells to accumulate around a cylindrical plug by a magnet under the culture plate (Figure 5D) (Yamamoto et al., 2009, 2010; Akiyama et al., 2010) or by using a ring-shaped magnet (Figure 5E) (Tseng et al., 2016).

Applications that require magnetic functionalization of cells by internalization of MNPs through endocytosis, cause concerns regarding potential cytotoxicicity of MNPs (Tomitaka et al., 2011). Several strategies have been suggested to reduce potential toxicity of magnetic functionalization. It was showed that compared to iron oxide MNPs, magnetoferritin as a biological MNP provided magnetic functionality provided with higher cell viability (Mattix et al., 2014a). In another study, Mattix et al. also presented a Janus structure of magnetic cellular spheroids with two distinct domains: cells and MNPs in the extracellular region (Mattix et al., 2014b). To assemble this structure, iron oxide MNPs, collagen and cell suspension were combined and magnetic cellular spheroids were formed by a hanging drop method. Study revealed that cellular internalization of MNPs in Janus magnetic spheroids is notably lower (35%) than uptake spheroids (83%), and that the Janus method contributed to better maintenance of spheroid viability (≥82%) in long-term cultivation. Janus magnetic spheroids were also successfully manipulated by an external magnetic field to assemble and fuse into a vascular tissue construct. Another approach to avoid the cytotoxicity of MNP internalization is cell surface engineering with MNPs. Dzamukova et al. described a cell surface engineering method based on the deposition of poly(allylamine)-stabilized MNPs on cell membranes without penetration to the cytoplasm (Dzamukova et al., 2015). The method did not affect membrane integrity or fundamental cellular functions (i.e., adhesion, proliferation, apoptosis) in either cancer or healthy cells and was used to generate layered cell sheets and 3D multicellular spheroids. It is also possible to manipulate label-free cells on a magnetic support. Lee et al. reported a magnetic cell levitation technique using iron-oxide encapsulated polymeric micro/nanoparticles as a substrate for 3D culture of tumor cells (Lee et al., 2011). Sugaya et al. described a manipulation method of cells and cellular spheroids via cell-size collagen hydrogel microbeads (Sugaya et al., 2012). Following the preparation of magnetic collagen hydrogel beads (> 20 μm) using microfluidic water-in-oil droplets, cells were attached to the collagen bead surfaces and cell-bead complexes were manipulated with a magnetic field.

Cells can be manipulated with a completely label-free principle based on negative magnetophoresis (Figures 5F–I) leading to a powerful alternative to eliminate acute and long-term cytotoxicity concerns due to cell binding or uptake of magnetic particles. Firstly, 3D cellular aggregates were formed by negative magnetophoresis using a paramagnetic medium (Akiyama and Morishima, 2011a). The experimental setup consisted of a culture chamber on four cubic NdFeB magnets (side dimension of 10 mm), placed with opposite poles next to each other. For cellular assembly, cells were suspended in a Gd-DTPA containing culture medium (34.6 mM) to enhance the diamagnetic property of the cells and poured into the chamber. Cells were aggregated into an egg-shaped structure in the center of the magnets, the area of lowest magnetic flux density, in 20 min. They also demonstrated another device for spheroid array formation that was able to form larger numbers of spheroids using the same principle (Akiyama and Morishima, 2011b). In this system, a cell culture chamber was set on a magnet array composed of 6 × 6 NdFeB magnets (3 × 3 × 10 mm), corresponding to 25 array spots with low magnetic flux densities at equal distance from each other. Furthermore, this magnetic cell manipulation principle was combined with microfluidic technology to generate rapid and high throughput systems for spheroid formation (Akiyama and Morishima, 2012). This microfluidic chip was composed of a cell aggregation chamber (8 mm in wide and 0.5 mm in deep) on a similar magnet array with 2 × 3 aggregation spots and a syringe pump for medium perfusion after aggregation. Cells assembled into spheroids within 1 min in the chip and most of the cells were alive in spheroids after 12 h of culture with medium perfusion. It is also possible to control the shape and size of cellular assemblies with the negative magnetophoresis principle. Abdel Fattah et al. placed two magnets, facing the same poles, on both sides of the cell culture chamber to form a rectangular bar-shaped cellular assembly and used also an arrangement of three magnets with 120° pole angles placed next to each other to form a three-pointed star-shaped cellular assembly (Abdel Fattah et al., 2016). Besides organizing cells into these different shapes, a strategy was developed that could control the size of spheroids generated by placing magnets under the cell culture chamber. This strategy involved the formation of larger spheroids by increasing the distance between the cell culture chamber and the magnet under the chamber, and consequently lowering the magnetic field strength (Abdel Fattah et al., 2016).

**Figure 6:**
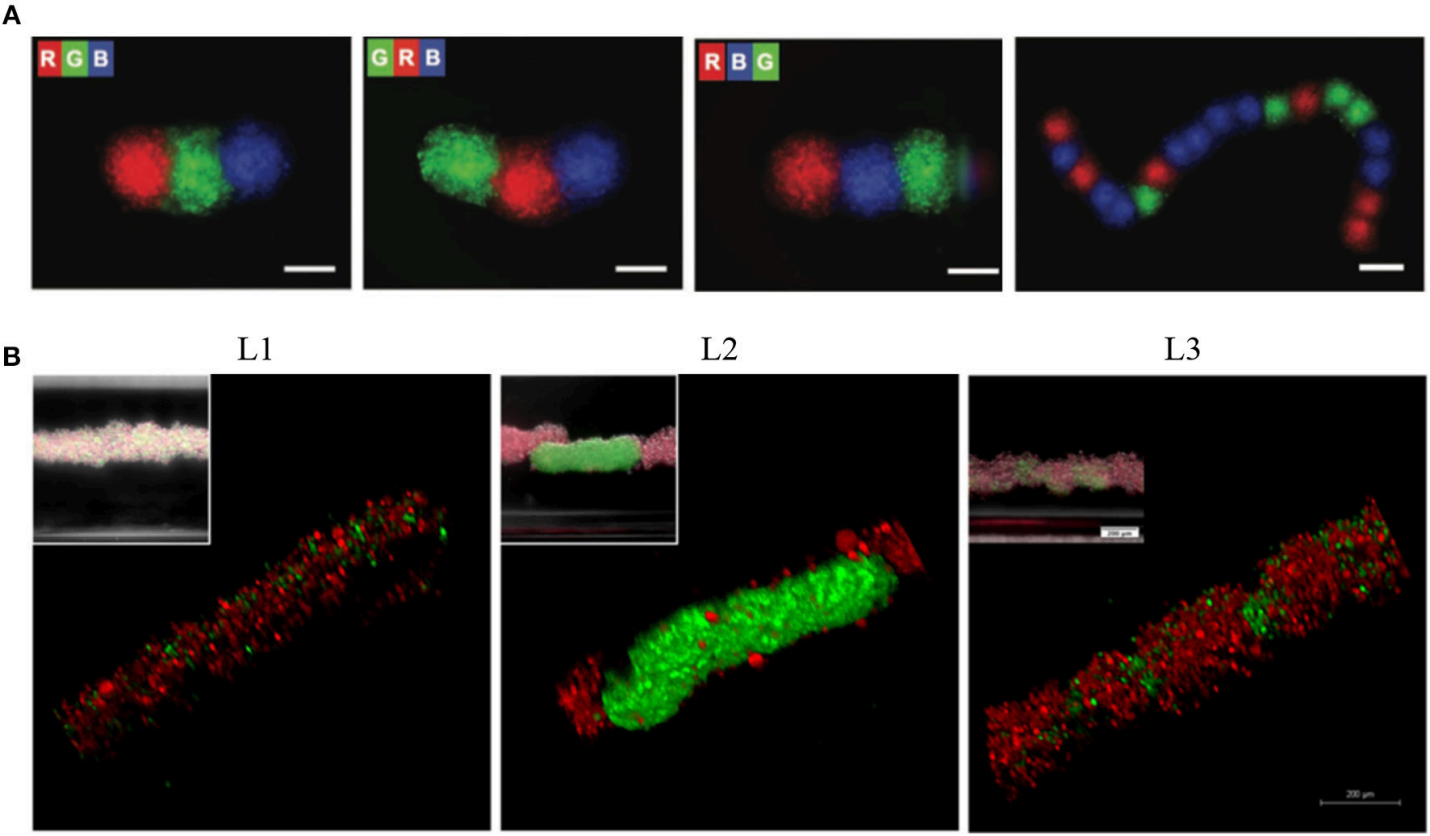
Levitation of diamagnetic cells with negative magnetophoresis; patterning of pre-formed spheroids into the tissue strings and *in situ* 3D cellular assembly. **(A)** Serial coding of spatially controlled spheroids. Spheroids were formed separately and then inserted into the levitation device (spheroids; R, red; G, green; B, blue) Scale bars, 100 μm. Reprinted from Tocchio et al. (2017). Copyright (2017) John Wiley & Sons, Inc. **(B)** Cellular assembly of D1 ORL UVA^eGFP^ and MDA-MB-231^dsRed^ cells under microgravity. Confocal and conventional fluorescence microscopy (upper left) images showing self-assembled coculture clusters formed with magnetic levitation (100 mM Gd-BT-DO3A) and different cell loading strategies; L1: simultaneously loading of MDA-MB-231^dsRed^ and D1 ORL UVA^eGFP^ cells, L2: MDA-MB-231^dsRed^ cells onto D1 ORL UVA^eGFP^ clusters formed with magnetic levitation and L3: D1 ORL UVA^eGFP^ cells onto MDA-MB-231^dsRed^ clusters formed with magnetic levitation (total 50,000 cells/culture chamber). Scale bars: 200 μm. Reprinted from Anil-Inevi et al. (2018).

Negative magnetophoresis is also a convenient technology for assembly of cells by a complete levitation of cells. Firstly, a system, composed of two permanent NdFeB magnets with the same poles facing each other and a container filled with a solution of paramagnetic ions between these magnets, was described for levitation of non-living materials (Mirica et al., 2009, 2010, 2011). Durmus et al. used a miniaturized magnetic levitation system, consisting of a glass capillary (with 1 mm inner diameter) between two NdFeB magnets to load cells in a paramagnetic medium, and two 45° tilted mirrors added to the sides to observe cells in real time, as a cell densitometry platform (Durmus et al., 2015; Tasoglu et al., 2015a). Following the study showing that the magnetic levitation system was suitable for the levitation of living cells, several studies aiming at manipulation of 3D living building blocks were conducted using this strategy. Cellular clusters of varying sizes were formed by changing the number of cells loaded into the capillaries within this system and fabricated tissue strings with patterning spheroids, already assembled with ultra-low attachment microplates (Figure 6A) (Tocchio et al., 2017). They also described a droplet-based magnetic levitation assembly design to form a larger number of 3D cellular structures. In this design, cells were compartmentalized in water-in-oil droplets generated by alternate aspiration of mineral oil and cell suspension, in the magnetic levitation device and assembled into individual 3D architecture within 24 h. This cellular assembly process resulted in faster cellular aggregation and enhanced shape uniformity of biological structures. As an alternative, Parfenov et al. designed a new installation consisting of 2 ring-shape NdFeB magnets (external diameter: 85 mm, internal diameter: 20 mm, thickness: 24 mm), oriented to each other with the same poles, a glass container (12 × 12 × 50 mm), inserted into the hole of the magnets and a camera system for label-free magnetic levitation of tissue spheroids (Parfenov et al., 2018). Besides the manipulation and fusion of the pre-formed 3D living structures, the manipulation of individual cells and formation of *in situ* 3D aggregates with controllable structures were also achieved in a magnetic levitation system (Anil-Inevi et al., 2018). In this study, the label-free magnetic levitation protocol was optimized by performing levitation of cells with several commercially available chelate forms and concentrations of gadolinium. A macrocyclic ligand containing the non-ionic form of gadolinium (gadobutrol) provided higher cell viability and higher levitation height. The optimal concentration (100 mM) of gadobutrol determined by considering the same evaluation criteria was found to be suitable for maintaining the viability of bone marrow stem cells cultured with magnetic levitation for 5 days. Furthermore, this protocol was shown to be convenient for the formation of larger cellular blocks up to ~2.7 cm in length, and for biofabrication and culture of various biphasic cellular structures consisting of bone marrow stem cells and breast cancer cells in a single magnetic levitation device (Figure 6B). It was also reported that fibroblast cells and lung cancer cells formed 3D structures and were cultured for 7 days using the magnetic levitation principle (Türker et al., 2018).

As cells can be manipulated without using MNPs, cells that form living structures with a desired shape and size in/on a support material can also be manipulated by magnetic forces. Tasoglu et al. first fabricated microscale photo-crosslinkable paramagnetic hydrogels with different sizes, assembled hydrogels in a controllable manner by manually moving the magnets above the liquid surface in a fluidic chamber and stabilized the resulting assembled gels by a secondary crosslinking step (Tasoglu et al., 2013). After all of the hydrogel fabrication and assembly stages, cell viability was still above 82%. In another study, microgels composed of photo-crosslinkable polymers (methacrylated gelatin or polyethylene glycol dimethacrylate) and laminin-coated microbeads were levitated in a paramagnetic medium within a magnetic setup composed of a reservoir between two permanent magnets with the same poles facing each other (Tasoglu et al., 2015b). Then, cell encapsulating hydrogels and cell seeded microbeads were used as building blocks for magnetic assembly within the device. They also described a method using an untethered magnetic micro-robot remotely controlled by magnetic fields for 2D and 3D manipulation of cell-encapsulating hydrogels (Tasoglu et al., 2014). In the system, motion of the magnetic microrobots (750 × 750 × 225 μm), polyurethane-encapsulated NdFeB particles, was guided by an electromagnet system surrounding the workspace and used to manipulate cellular building units.

#### 2D and 3D Cellular Patterning

Patterning is an important tool to mimic natural homotypic or heterotypic cellular arrangements for cell biology and tissue engineering applications (Guillotin and Guillemot, 2011). Precise spatiotemporal control and guidance of individual cells via micromanipulation techniques have great potential to provide convenient cellular microenvironments and accurately imitate *in vivo* chemical and physical cues (El-Ali et al., 2006). It is possible to pattern both adherent and suspension cells in the culture. Patterning of adherent cells is conventionally performed via material surface modification with cell-adhesion ligands to guide cells toward adhesive areas (Veiseh et al., 2007). For the patterning of suspension cells, tools that generate an external force such as a strongly focused beam of light (Grier, 2003), an electric field (Matsue et al., 1997) or a magnetic field (Ino et al., 2007) are used. Among all these techniques, the patterning of cells with magnetic force provides minimized interference with the biochemical functions of cells (Lai et al., 2010). Firstly, cells labeled with magnetite cationic liposomes (MCLs) were manipulated to form curved, parallel, or crossing patterns through a setup, composed of steel plates (L: 30 mm; H: 2 mm; T: 200 μm) on a magnet (Ino et al., 2007). They also patterned HUVECs on Matrigel to form cord-like structures. Using a similar technology, line patterning of HUVECs was formed on monolayer cells, skin tissues and different types of cell sheets (Akiyama et al., 2009). Furthermore, they achieved incorporation of patterned HUVECs into layered myoblast sheets. To generate these 3D constructs, magnetic accumulation of myoblasts into cell sheets and magnetic patterning of HUVECs on each sheet layer were alternated. Fujita et al. demonstrated that the magnetic cell pattern could be used to produce scaffold-free contractile skeletal muscle (Fujita et al., 2010). In the study, magnetite-incorporated myogenic cells were patterned linearly on a monolayer of fibroblast cells, using a magnetic field concentrator. The tendon structure was modeled by two collagen films that were fixed on a culture dish. Alignment of myotubes was enhanced through linear patterning of the cells and fibroblast monolayer prevented the construct shrinkage. Furthermore, construct had the presence of sarcomere structures, expression of muscle proteins and the exhibition of active tension (~1 μN) when stimulated with electric pulses.

Magnetic force-based cell patterning technology also allows studies related to the behavior of individual cells. Ino et al. developed single cell culture arrays composed of a pin holder (soft iron) with more than 6,000 pillars (100 × 100 × 300 μm) placed on a magnet to concentrate the magnetic flux density on the culture dish areas right above the pillars (Ino et al., 2008). MCL-labeled cells were allocated on the pillars and single cell patterning was obtained when the number of cells seeded was sufficiently reduced. Cellular aggregates were also successfully collected from the culture using a micromanipulator. In addition, the same system was used to investigate cell–cell interactions between HUVECs (Ino et al., 2009). Following the demonstration that the labeling of cells with MCLs had no effect on the viability and function of HUVECs, they changed the distance of the cellular aggregates on spots (center-to-center distance: 250 and 350 μm) using magnetized pin holder devices with different configurations and showed the effect of distance between cells on tubular formation. This magnetic patterning system also allowed for analysis of the invasive capacity of cancer model cells. Magnetically-labeled BALB/3T3 mouse fibroblast cells transformed with *v-Src* were seeded into a culture dish with a thin layer of collagen gel (Okochi et al., 2009). After patterning of cells into an array by placing the culture dish on a magnetized pin holder device (center-to-center distance: 250 μm), cells were further embedded with collagen gel to form the 3D cell array. For the culture of 3D aggregates, the magnetized pin holder was removed from the culture dish and this *in vivo*-like 3D model was used to investigate the invasive capacity of cells under different conditions. Another cell patterning method was presented for biochip applications (Ger et al., 2013). Cell culture arrangements were achieved through diamond-shaped magnetic thin films (size of each diamond: 90 × 50 μm). When an external magnetic field was applied along the long axis of the thin film, the magnetically-labeled cells were successfully pulled toward the tips of the diamond-shaped thin film structure and formed a linear pattern.

An alternative approach for cell patterning is hydrogel-based magnetic cell patterning techniques. Fu and co-workers described a patterning method based on magnetic force and photoresponsive hydrogels (Fu et al., 2011). Hydrogels were magnetized with magnetic particles, and magnetic hydrogel blocks with the desired pattern were fabricated by photolithography. First, magnetic blocks were pulled toward the cell adhesion surface with a magnet and the first type of cells were seeded to adhere to the region not covered by the hydrogel. To form heterotypic cell pattern, the hydrogel on the surface was removed using a magnetic probe and the second type of cells was seeded to adhere to the empty area on the culture surface. Grogan et al. reported another magnetic cell patterning technique that relied on the alignment of magnetically-labeled cells in hydrogel by specifically orientated external magnetic fields (Grogan et al., 2012). Cellular arrangements were altered by manipulating the strength of the magnetic field, distribution of field lines and using calcium chloride crosslinking gradients in alginate hydrogels. Magnetically-labeled cells in this tissue engineering structure could be monitored using magnetic resonance imaging *in vivo*.

Besides manipulation and patterning of individual cells, patterning of spheroids is critical to form complex tissue-mimicking structures. Lin et al. presented an organoid patterning apparatus composed of a magnetic field–defining steel construct, that was fabricated by machining according to the targeted patterns on a permanent magnet and patterned preformed magnetically-labeled spheroids into rings, lines, and arrays (Lin et al., 2008). Stem cell spheroids were also patterned into various configurations (circle, distinct islands, four-leaf clover or line) to enable the study of fundamental principles in developmental biology (Bratt-Leal et al., 2011). In the study, cells were labeled by physical entrapment of magnetic particles in the extracellular space of spheroids during formation to allow assembly without directly perturbing intracellular processes. Magnetically guided patterning of spheroids were also achieved using functionalized super paramagnetic iron oxide nanoparticles (SPIONs) to label cells with enhanced cytocompatibility (Whatley et al., 2014). The desired patterns were obtained through magnetic templates fabricated from magnetic sheets using a computer controlled cutting device.

Although most of the applications for magnetic force-based patterning of cells or cellular spheroids have been achieved by positive magnetophoresis, based on magnetic labeling of cells, they can also be patterned in a label-free manner by negative magnetophoresis. It was shown that HUVEC cells that were suspended in an inert and biocompatible ferrofluid were aligned parallel to the applied magnetic field and the linear cellular structures were stable even after removal of the ferrofluid and magnetic field (Krebs et al., 2009). Tocchio et al. levitated and patterned preformed spheroids with more than 15 repeating units in a paramagnetic medium through magnetic field gradient between two permanent magnets placed on the top and bottom of a culture capillary channel (Figure 6A) (Tocchio et al., 2017). To achieve spheroid-to-spheroid contact and thus formation of tissue string with the desired order, spheroids were successively inserted into the capillary and the levitation setup was temporarily tilted for ~5 min. Although patterning of cells with negative magnetophoresis eliminates the drawbacks arising from cell labeling, it limits the flexibility of the pattern variety as the cell or spheres are directed toward the area where the magnetic field is low and this area cannot be controlled easily.

#### Other Applications

Magnetic cell manipulation techniques can be used for further auxiliary purposes in cell culture systems for various tissue engineering applications and study of fundamental biological principles in a controlled manner. One of these applications is cell sheet engineering, that aims to keep cells and ECM together via formation of living sheet structures and thus to constitute *in vivo*-like models for various specific tissues (Yamato and Okano, 2004; Yang et al., 2005). Although the formation of tissues by combining spheroids as building blocks is a promising approach to construct complex structures, it has been shown that thin tissue sheets composed of cell layers are also functional and useful for some applications such as tissue engineering in heart (Matsuura et al., 2014) and cornea (Umemoto et al., 2013). Cell sheet engineering that can maintain the intact cell matrix provides a convenient microenvironment for vascularization. Magnetic force-based cell sheet engineering eliminates the need for thermosensitive surfaces that are used in conventional methods to form cell sheets.

It was reported that magnetic force-based manipulation of cells could be used to construct and harvest keratinocyte sheets (Ito et al., 2004a). MCL-labeled human keratinocytes were cultured in a low-attachment plate with a cylindrical NdFeB magnet positioned under the plate to create a magnetic force vertical to the plate. After 24 h of culture, 5-layered keratinocyte sheets were formed, and keratinocytes were further stratified into 10-layered epidermal sheets by additional cultivation in high-calcium medium. This technology was also used to construct a heterotypic, layered co-culture system of hepatocytes and endothelial cells (Ito et al., 2004b) or mesenchymal cells (Ito et al., 2007) to obtain tight and close cellular contact. This tight layered co-culture structure exhibited enhanced albumin secretion by hepatocytes compared to homotypic culture or heterotypic co-cultures produced without using magnetic manipulation of cells.

Retinal pigment epithelium (RPE) cells present another example that needs formation of sheet-like structures due to microscale transplant requirements. Magnetically-labeled human RPE cells were guided into sheets and 15-layered cell sheets were formed after 24 h of culture via the same magnetic manipulation technique (Ito et al., 2005a). Furthermore, magnetic force-based cell sheet engineering technology was used for bone tissue engineering (Shimizu et al., 2007c). In this work, magnetically-labeled mesenchymal stem cells (MSCs) were formed into multilayered sheet-like structures after 24 h of magnetic guidance, the sheets were then differentiated into osteoblasts for 21 days. Differentiated sheets were transplanted into the bone defect in the crania of nude rats and new bone formation in the defect area was observed in 2 weeks after the transplantation. Magnetic cell sheet engineering was used in a regenerative medicine strategy for ischemic heart disease (Ishii et al., 2014). Isolated mouse adipose-derived regenerative cells (ADRCs) were labeled with MCLs, mixed with an ECM precursor solution and cultured in an ultra-low attachment plate with a magnet placed underneath the plate to accumulate ADRCs at the bottom of the culture. Multilayered cell sheets formed after 24 h of culture, which were subsequently transplanted onto the infarcted myocardium and resulted in functional and structural improvements in ischemic hearts. Similarly, magnetite tissue engineering technology was used to create induced pluripotent stem (iPS) cell sheets for reparative angiogenesis, and revascularization was shown to be promoted after engraftment of engineered sheets into the ischemic tissues of nude mice (Kito et al., 2013). In addition to these studies that illustrate the unmodified use of cellular sheets, preformed magnetic cell sheets can also be magnetically directed to the desired macrostructures. Ito et al., for example, rolled a cylindrical magnet in a silicone tube over the cell sheet to form a cellular tube (Ito et al., 2005b).

Another application of magnetic cell guidance in cell culture is magnetic force-based cell seeding into scaffolds. The complex architecture of scaffolds can cause technical difficulties in cell seeding and this results in a non-uniform and inadequate migration of cells into the depth of the structure (Melchels et al., 2010). Shimizu et al. seeded magnetically-labeled fibroblasts on varying pore size scaffolds with with a magnet (400 mT) positioned under the scaffold (Shimizu et al., 2006). Presence of magnetic forces improved cell-seeding efficiency for all scaffold types. Cell seeding efficiency was further increased when a high-intensity magnet (1,000 mT) was used. Shortly after, the same group increased seeding efficiency of magnetically-labeled fibroblasts onto porcine decellularized common carotid artery (dCCA) by inserting a cylindrical magnet into the lumen of dCCA (Shimizu et al., 2007a). System not only increased the number of cell attached to the scaffold, but also enhanced infiltration and distribution (Thevenot et al., 2008). Magnetic cell seeding technique was also applied to bone tissue engineering using bone marrow stromal cells and 3D hydroxyapatite scaffolds and cultured cells in the osteogenic induction medium for 2 weeks (Shimizu et al., 2007b). The results indicated that cells seeded with magnetic cell manipulation expressed higher levels of osteogenic markers than cells seeded with static technique. Although studies on the use of magnetic cell manipulation for cell seeding on the scaffolds have so far aimed at the formation of large living structures, this technique has potential to be used to direct cells into desired regions of the 3D structures in miniaturized systems.

## Discussion and Conclusion

Sorting rare cells with high purity provide a huge application potential in medical pursuits including regenerative medicine, personalized therapy as well as diagnostics (Tanaka et al., 2009; Toss et al., 2014; Kumar et al., 2017). Classical cell separation methods include membrane-based filtration and centrifugation, and have been applied to the separation of clinically relevant cells such as tumor cells (Vona et al., 2000) and bone marrow (BM) micrometastatic cells in cancer patients (Choesmel et al., 2004). However, filtration, in general, is limited by the pores of the membrane that are prone to clogging. Centrifugation, on the other hand, may induce phenotype affecting shear stresses on cells (Autebert et al., 2012). Moreover, these two techniques lack automation and not suitable for single cell operations. Modern tools such as fluorescence-activated cell sorting (FACS) (Picot et al., 2012) and MACS (Grützkau and Radbruch, 2010) offer more powerful, robust and high-throughput cell sorting. In FACS, cells labeled with antibody-conjugated fluorescence molecules are identified via fluorescence signal coming from each cell in a flow cytometry system. But FACS requires sophisticated and expensive machines. Besides, direct separation of rare cells from whole blood is not applicable using conventional FACS. MACS, which requires labeling of cells with magnetic particles and subsequent flow through high magnetic gradient columns, is a rather simple and inexpensive method compared to FACS. However, it shows an insufficient sensitivity when applied to low abundance subpopulations in the sample (Leary et al., 2002). These limitations have led the development of microfluidic devices that could allow cost-effective, sensitive, and high-throughput separation and subsequent analysis of different cell types.

Active cell sorting devices which use external forces for cell manipulation are comprised of optical, dielectrophoretic, acoustic, and magnetic fields (Bhagat et al., 2010). Optical platforms are the least portable since they require an optic system and a high-power laser source (Wang et al., 2004). Dielectrophoresis (DEP) represents a good alternative if target cells have distinct intrinsic electric properties (Pohl and Hawk, 1966; Pohl and Crane, 1971). However, DEP may cause Joule heating that harm cells suspended in a culture medium that have high electric conductivity (Tang et al., 2014). Acoustophoresis (ACP) uses sound waves to manipulate cells based on their size, density and compressibility (Petersson et al., 2005). Compared to DEP, ACP affects cell viability less (Hultström et al., 2007) and has been used to separate CTCs from WBCs (Li et al., 2015). However, compressibility of the many rare cells is not well-documented. One of the assets of magnetic techniques over its counterparts is that the separation could be achieved with minimal damage to cells (Kim et al., 2013b). Besides, simplicity of inserting an inexpensive permanent magnet or an electromagnet makes magnetophoresis more suitable for on-chip/microfluidic separation applications. In contrast to DEP and ACP, magnetophoresis may include the use of magnetic labels which are rather advantageous compared to fluorescent labels since they are more stable and do not require light excitation (Hahm, 2011).

A wide range of basic and advanced techniques has been currently available to assemble cells into 3D structures. Conventional methods of cellular assembly into 3D, such as cell hanging drop (Timmins et al., 2004) and liquid overlay (Lei et al., 2017) are labor intensive, time consuming and only allow low throughput. The methods that enhance throughput, such as spinner culture (Nyberg et al., 2005) and rotating cell culture (Nishi et al., 2013), cause loss of control over the size and uniformity of the generated 3D living structures. Alternatively, external forces can also be employed to assemble cells into 3D via dielectrophoresis (Voldman, 2006), acoustophoresis (Petersson et al., 2007; Bouyer et al., 2016), and magnetophoresis. Magnetophoresis retain the advantages it presents for cell separation in 3D assembly of cells as well, involving cell health, instrumentation, and cost.

Patterning of cells for 2D or 3D culture currently can be carried out by chemical, physical, or combinatorial techniques. Surface chemistry-based methods provide a high precision way to collocate cells on surfaces in predesigned patterns taking advantage of recognition-based cell adhesion (Ogaki et al., 2010). However, the need for a pre-treated surface requires additional steps such as the fabrication and characterization of the surface. In addition, these methods can be applied only for surface-dependent cell cultures. Although this limitation can be surpassed by guiding cells by cell-repellent interfaces, the application loses recognition-based high precision. Physical guidance of cells into targeted patterns is possible via dielectrophoresis, acoustophoresis, and alternatively bioprinting techniques, such as inkjet printing (Xu et al., 2013) and laser-based techniques (Schiele et al., 2010). Precise cell patterning applications without cellular damage by bioprinting techniques require large instrumentations and complex set-ups. Magnetophoresis eliminates the need for these complex instrumentations and offers nozzle free arrangement of the cells in the desired organization without affecting their viability and function.

Despite the advantages of the magnetic force-based cell manipulation methods reviewed here, the biological effects of its components acting on biological systems should not be overlooked, especially for long-term cell culture. The magnetic field, which is an important component of magnetic-based cell manipulation systems in both positive and negative magnetophoresis, can have different effects on living cells. The impact of the magnetic field on cells depends on magnetic field intensity (Zhang et al., 2014, 2016), type (static or dynamic) and spatial distribution of magnetic fields (homogeneous or inhomogeneous) (Zhang et al., 2017c), exposure time (Sullivan et al., 2011), cell type and density (Zhang et al., 2017b), and cellular infection (Nam et al., 2013). Several cellular processes are influenced by the magnetic field, such as membrane properties (Lin et al., 2013), cell shape and cytoskeletal organization (Chionna et al., 2005), cell cycle (Mo et al., 2013), cell viability and proliferation (Wang et al., 2016a; Maredziak et al., 2017), cell orientation (Kim et al., 2008a), cell adhesion, migration (Mo et al., 2016) and differentiation (Zhang et al., 2014). Evidence from several studies has shown that the biological effects of magnetic fields are correlated with their intensity. While weak or moderate magnetic fields (<1 T) have only slight effects on cells (Glade and Tabony, 2005; Zhang et al., 2016), strong magnetic fields (>20 T) can have drastic effects such as altering mitotic spindle orientation (Zhang et al., 2017a). Despite the fact that the magnetic field used for cell manipulation is usually weak or moderate, the magnitude of the applied magnetic field and the duration of the magnetic field exposure must be considered as these factors may affect the success of the application depending on cell type.

An important component of negative magnetophoresis is magnetic liquids, i.e., ferrofluid (Zhu et al., 2010, 2012), or paramagnetic salt solution (Shen et al., 2012; Abdel Fattah et al., 2016). Magnetic liquids are needed to increase the repulsive magnetic forces on diamagnetic cells through enhancing medium magnetic susceptibility and thus eliminate problems arising from the use of high magnetic field exposure. However, the biocompatibility of magnetic liquids is an important and challenging issue for tissue engineering applications. Studies that were carried out to understand the biocompatibility of paramagnetic salt solutions and ferrofluids, are summarized in Table 3. Although the effect of magnetic fluids on cell viability is under investigation, and some biocompatible magnetic fluids have been reported, other cellular influences should be considered specifically depending on application.

**Table 3:** Biocompatibility of magnetic liquids.

**Magnetic liquids**	**Structures**	**Short-term biocompatibility**	**Long-term biocompatibility**	**References**
Gadolinium diethylenetriaminepentaacetic acid	Linear ionic	NR	+	Winkleman et al., 2004
		++	NR	Rodríguez-Villarreal et al., 2011
		++++	NR	Abdel Fattah et al., 2016
Gadoteridol	Macrocyclic nonionic	NR	+++	Kauffmann et al., 2011
Gadabutrol (Gadavist®)	Macrocyclic nonionic	NR	++++	Durmus et al., 2015; Anil-Inevi et al., 2018
		NR	+++	Tocchio et al., 2017
Gadodiamide (Omniscan™)	Linear nonionic	NR	++	Anil-Inevi et al., 2018
Gadopentetate dimeglumine (Magnevist®)	Linear ionic	NR	+++	Anil-Inevi et al., 2018
Gadoterate meglumine (Dotarem®)	Macrocyclic ionic	NR	++	Kauffmann et al., 2011
		NR	+++	Anil-Inevi et al., 2018
Gadobenate dimeglumine (Multihance®)	Linear ionic	NR	±	Kauffmann et al., 2011
		NR	+++	Anil-Inevi et al., 2018
BSA (bovine serum albumin) coated ferrofluid	Globular protein-magnetite nanoparticles	±	NR	Krebs et al., 2009
Citrate stabilized ferrofluid	Citrate anion-Cobalt-ferrite nanoparticles	–	NR	Kose et al., 2009
EMG 408 ferrofluid	Anionic surfactant- magnetite nanoparticles	++++	NR	Zhu et al., 2012
Graft copolymer functionalized ferrofluid	Nonionic polymers-maghemite nanoparticles	++++ ++++	NR	Zhao et al., 2015
		++	NR	Zhao et al., 2017a,b

*Cell viability which was not statistically different from the control group, or above 80% was assessed as good cell viability. Good cell viability levels were scored as– (< 10 mM), ± (10–25 mM), + (25–50 mM), ++ (50–100 mM), +++ (100–200 mM) or ++++ (>200 mM) for paramagnetic salt solutions. Good cell viability in ferrofluids were scored as– (good cell viability: < 0.06%, volume fraction of magnetic particles), ± (0.06–0.12%), + (0.12–0.25%), ++ (0.25–0.5%), +++ (0.5–1%) or ++++ (>1%). NR stands for “not reported,” respectively*.

Positive magnetophoresis-based cell manipulation using magnetic labels usually offers high purity, selectivity and recovery rate for cell separation and high manipulation flexibility for guidance of cells into pre-designed organizations in 2D and 3D cell culture. However, it should be noted that long-term retention of magnetic labels might affect cellular function, viability and phenotypic characteristics of some sensitive cell populations such as stem cells and progenitor cells (Farrell et al., 2008; Mahmoudi et al., 2011; Plouffe et al., 2015). In addition, internalization of small particles (<100 nm) creates reactive oxygen species (ROS) (Soenen and De Cuyper, 2010), which could damage the structure and function of cellular components when produced at elevated levels (Sharifi et al., 2012; Liu et al., 2013). In cell culture applications, although cellular alterations arising from MNPs internalization may be minimized by several strategies such as the usage of biological MNPs and forming Janus structure, these methods also require different additional steps that need to be well characterized, and still time-consuming. It is also challenging to detach MNPs after separation (Hosic et al., 2016). But one of the biggest challenge regarding rare cell separation in clinical settings is selecting the target cells baring several surface markers from an initial bulk of cells (Plouffe et al., 2015). In case of CTCs that show genetic and phenotypic heterogeneity (Baccelli et al., 2013; Yu et al., 2013), surface marker-based magnetic detection and isolation techniques become challenging. At this point, negative magnetophoresis-based label-free cell manipulation strategies becomes an attractive option. Nevertheless, this technique has been scarcely exploited for microfluidic magnetic sorting of key rare cells such as stem cells and tumor cells (Table 1). Improvements in negative magnetophoresis could open a new window for accurate and high-throughput handling of rare cells based on cells' intrinsic properties.

Lately, the use of magnetic forces has become a favorable approach in miniaturized microfluidic systems that offer minimal size, cost, and analysis time compared to complex and expensive laboratory equipment and also enhance magnetic flux density with magnetizable on-chip micropatterns (Tekin and Gijs, 2013; Shields et al., 2015). Nevertheless, microfluidic cell sorting systems usually have lower throughput compared to FACS system (50,000 cells s^−1^) and therefore require further improvement (Dharmasiri et al., 2010). In this context, attempts to increase the throughput of the microfluidic systems, such as chip parallelization could be implemented (Hosic et al., 2016). So far, many of the works related to magnetically-guided cell separation were proof-of-principle demonstrations of the systems and designs with low medium complexity or user defined mixtures (e.g., biological samples spiked with cancer cells). Even though pioneering results were reported, each concept should be validated using complex cell suspensions (e.g., whole blood) to be successfully translated to real-world applications in near future. Magnetic manipulation is performed by permanent or electromagnets which are either placed outside or integrated into the chip. Obtaining a desired magnetic field gradient at a certain location within the microfluidic platform is necessary for repeatable results. For delicate control of magnetic fields in microfluidic channels, external magnets should be aligned to the channel with high-precision. Integrated micromagnets on microfluidic channel provides enhanced control of magnetic field. However, these micromagnets require costly microfabrication steps (Pamme, 2006).

In addition to obtaining the cell subpopulation of interest from a bulk population, magnetic cell manipulation is a crucial tool to guide cells into the natural-like structures for culture. The future of 2D and 3D cell culture for bottom-up tissue engineering applications, fundamental biological and pharmaceutical research lies in the generation of complex cellular organizations in a spatiotemporally controllable manner, and magnetic force-based methods provide a relatively new and precise contactless cell manipulation with minimized biological effects. Moreover, the mechanical support-free nature of the technology provides an opportunity to eliminate the biological effects of the support material. Although most studies have involved application of magnetic cell guidance on a macroscale, recent efforts have focused on combining magnetic force-based cell manipulation with microfluidic technology for creation of concentrated and strong magnetic forces with inexpensive setups, more precise control of cellular interactions and dynamic microenvironment, reduced consumption of reagents, and efficient high throughput experimentation. The recent applications of this concept involve using magnetic levitation technology as a label-free biofabrication method (Tocchio et al., 2017; Anil-Inevi et al., 2018; Türker et al., 2018). This label-free magnetic cell guidance technique has great potential in several exciting applications. Recent research shows that cellular building blocks can be fused in a nozzle-free manner to produce functional and large-sized tissues (Parfenov et al., 2018), and this progress presents a preview of the immediate future for application of magnetic-based cell manipulation in tissue engineering.

Among the applications of magnetic levitation, ground-based facilities for simulation of microgravity through magnetic levitation (Qian et al., 2013) must be mentioned due to their promise to improve our understanding of the biological effects of microgravity as an alternative to expensive and rare spaceflight experiments. Not all magnetic levitation technologies are suitable for this purpose. Specifically, magnetic levitation by labeling diamagnetic cells with magnetic particles cannot properly simulate microgravity owing to the fact that magnetic force cannot be applied to all cellular units homogeneously (Souza et al., 2010; Haisler et al., 2013). On the contrary, magnetic levitation of diamagnetic cells by negative magnetophoresis creates forces acting on all cellular structures. While other ground-based techniques to simulate microgravity, such as the rotating-wall vessel (RWV) platform (Rucci et al., 2002), 2D clinostats (Qian et al., 2012), and Random Positioning Machines (RPM) (Wuest et al., 2015), generate fluid shear stress on the cells as a result of rotation and interrupts the cellular response (Pavalko et al., 1998; Kaysen et al., 1999), the magnetic levitation principle does not create such additional forces on the cells. Furthermore, magnetic levitation presents an alternative to the use of earth-based animal models (Ozcivici et al., 2007, 2010; Ozcivici and Judex, 2014), which are limited by expensive setups, high variation at the molecular/cellular level, and ethical concerns. In conclusion, magnetic levitation is a powerful technique that can potentially be applied to test unique hypotheses in gravitational biology and tissue engineering research.

In this review, we highlighted magnetofluidic cell manipulation applications including rare cell separation and 2D and 3D cell culture. Although some improvements in throughput, purity and viability are required for current techniques, the rapid growth in the field could spread magnetofluidic-based rare cell separation tools from the research laboratory to industry in the future. Furthermore, progressive development in magnetic cell manipulation techniques and recent attempts for integration of these techniques with microfluidic technology represent exciting tools in near future for more complex and precise tissue engineering and cell biology research. Based on the studies highlighted in this review, we envision that the future trend of magnetic force-based microfluidic systems will be toward the development of label-free cell separation platforms that perform series of all necessary tasks (such as filtration, separation, and analysis of cells) on a single chip and the improvement of cell culture systems for scaffold- and nozzle-free biofabrication of 3D living constructs, simulation of microgravity and simultaneous monitoring of drug effects.

## Author Contributions

EO and HCT designed the content of the article; all authors performed literature survey and wrote the article. SY and MA-I prepared the figures and tables. EO and HCT edited and reviewed the article before submission.

### Conflict of Interest Statement

The authors declare that the research was conducted in the absence of any commercial or financial relationships that could be construed as a potential conflict of interest.
