# Supplementary material for: Magnetic Force-Based Microfluidic Techniques for Cellular and Tissue Bioengineering
Source: Front Bioeng Biotechnol. 2018 Dec 19;6:192. doi: 10.3389/fbioe.2018.00192 (PMC6305723; doi:10.3389/fbioe.2018.00192)
Supplement: Supplementary file 1 [file Data_Sheet_1.PDF]

SUPPLEMENTARY INFORMATION

Magnetic Force-Based Microfluidic Techniques for Cellular and Tissue Bioengineering

Sena Yaman, Muge Anil-Inevi, Engin Ozcivici*, H. Cumhur Tekin*

Department of Bioengineering

Izmir Institute of Technology

Urla, Izmir, Turkey

*equal correspondence

* Correspondence to:

Engin Ozcivici, Ph.D.

Department of Bioengineering Izmir Institute of Technology

Urla, Izmir, 35430

Turkey

Phone: + 90 232 750 7385

Fax: + 90 232 750 7603

e-mail: enginozcivici@iyte.edu.tr

H. Cumhur Tekin, Ph.D.

Department of Bioengineering Izmir Institute of Technology

Urla, Izmir, 35430

Turkey

Phone: + 90 232 750 7388

Fax: + 90 232 750 7603

e-mail: cumhurtekin@iyte.edu.tr

Physics of Magnetic Force-Based Manipulation

The general equation for the magnetic force acting on a particle (or cell) is given by Eq. 1 (Weston et al., 2010):

$$\overrightarrow{F_M} = (\vec{m}.\nabla)\vec{B} \tag{1}$$

,where B is the magnetic flux density (in Tesla, T), and ∇ is the del operator. $\vec{m}$ is the magnetic dipole that takes the form of Eq. 2 in low magnetic fields (Pamme, 2006; Gijs et al., 2010):

$$\vec{m} = \frac{V.\Delta\chi}{\mu_o}\vec{B} \tag{2}$$

,where μ_o is the permeability of free space which is 1.2566 $\times 10^{-6}$ kg·m·A^{-2}·s^{-2}. Therefore, the magnetic force acting on a particle depends on the volume of the particle (*V*), the magnetic susceptibility difference *(*$\Delta\chi$*)* between the particle (χ_p) and the surrounding medium (χ_m), together with the gradient and strength of the magnetic flux density. The force equation can be rearranged as:

$$\overrightarrow{F_M} = \frac{V.(\chi_p - \chi_m)}{\mu_0}(\vec{B}.\nabla)\vec{B} \tag{3}$$

For a Cartesian coordinate system, $(\vec{B}.\nabla)\vec{B}$ is expanded into:

$$(\vec{B}.\nabla)\vec{B} = \begin{pmatrix} B_x\frac{\partial B_x}{\partial x} + B_y\frac{\partial B_x}{\partial y} + B_z\frac{\partial B_x}{\partial z} \\ B_x\frac{\partial B_y}{\partial x} + B_y\frac{\partial B_y}{\partial y} + B_z\frac{\partial B_y}{\partial z} \\ B_x\frac{\partial B_z}{\partial x} + B_y\frac{\partial B_z}{\partial y} + B_z\frac{\partial B_z}{\partial z} \end{pmatrix} \tag{4}$$

During magnetic manipulation of particles in the medium, the Stokes' drag force (F_D) appears in the opposite direction of the particle movement which is given for a spherical object as follows (Bhuvanendran Nair Gourikutty et al., 2016):

$$\overrightarrow{F_D} = 6\pi R\eta f_d(\upsilon_p) \tag{5}$$

Here R is the particle radius, η is the dynamic viscosity of the surrounding medium, f_d is the drag coefficient and υ_p is the velocity of the particle. Inertial effects are usually neglected due to small Reynolds numbers in microfluidic magnetophoresis. Therefore, dominant forces for the motion of a particle in medium can be expressed by Eq. 6 (Hejazian et al., 2015):

$$m\frac{\mathrm{d}\overrightarrow{\upsilon_p}}{\mathrm{dt}} = \overrightarrow{F_M} + \overrightarrow{F_D} + \overrightarrow{F_G} + \overrightarrow{F_B} \tag{6}$$

Here m is the mass of the particle; $\overrightarrow{F_G}$ is the gravitational/buoyant force, which is calculated as follows:

$$\overrightarrow{F_G} = V\Delta\rho g \tag{7}$$

with the volumetric density difference ($\Delta\rho$) between the particle (ρ_P) and the medium (ρ_M), and the gravitational acceleration g (9.8 m·s^{-2}).

For the particles with sufficiently small diameters (Gerber et al., 1983) (i.e. down to a few tens of nanometers), the Brownian force, $\overrightarrow{F_B}$, becomes significant in the balance (Eq. 6) and particle diffusion due to Brownian motion influences the magnetic manipulation.

In case of magnetic levitation, the particles placed between two opposing magnets levitate at a unique position where the magnetic force is balanced by the gravitational force (Durmus et al., 2015):

$$\overrightarrow{F_M} + \overrightarrow{F_G} = 0 \tag{8}$$

$$\frac{V.(\chi_p - \chi_m)}{\mu_0}(\vec{B}.\nabla)\vec{B} = V.(\rho_P - \rho_M)\, g \tag{9}$$

As demonstrated by Eq. 8, equilibrium position of a particle (or cell) is independent of the volume (V) during magnetic levitation.

The behavior of a particle under an external magnetic force is determined by the magnetization (or magnetic susceptibility (χ)) of that particle. Particles and cells show either

diamagnetic, paramagnetic or ferromagnetic behavior. "Diamagnetic" particles have negative magnetic susceptibility values (-10^{-6} to -10^{-3}) (e.g. DNA, proteins, cells, water). They do not have magnetic dipoles within the material in the absence of an external magnetic field. Upon the application of the external magnetic field, a magnetic dipole which is aligned antiparallel to that field is created. Therefore, diamagnetic particles are repelled by the magnetic field and tend to move to the area in which the magnetic field strength is minimum. "Paramagnetic" particles have positive susceptibility values on the order of 10^{-6} to 10^{-1} (e.g. gadolinium, magnesium, titanium, oxygen, aluminum, iron oxide), and have magnetic dipoles in the unmagnetized state (Sun et al., 2008; Kolhatkar et al., 2013). Magnetic dipoles in paramagnetic materials only align parallel in the magnetized state. Therefore, under the external magnetic field, particles move towards to the maxima of the magnetic field strength. Upon removal of the magnetic field, they do not retain any magnetization (Kolhatkar et al., 2013). On the other hand, "ferromagnetic" particles (e.g. such as iron, nickel, and cobalt) have large magnetic susceptibility values, and aligned magnetic dipoles even though there is no external magnetic field (Pankhurst et al., 2003; Issa et al., 2013). In the presence of an external magnetic field, they are subjected to a strong attractive force along the direction of the field and retain magnetization to some extent after the removal of the magnetic field (Gijs, 2004; Issa et al., 2013). Moreover, ferromagnetic particles are constituted of structures of magnetic domain. A magnetic domain is a region in which all magnetic dipoles are oriented in the same direction (Akbarzadeh et al., 2012). Therefore, a single-domain ferromagnetic particle possesses magnetization in a uniform direction. "Superparamagnetism" is a phenomenon in which the size of a single-domain particle is reduced below a certain critical diameter so that the magnetic moments flip direction randomly in response to thermal energy (Bean and Livingston, 1959; Brown, 1963; Pankhurst et al., 2003). At temperatures above the so-called blocking temperature (T_B), superparamagnetic particles exhibit paramagnetic behavior with larger magnetic susceptibilities than those of typical paramagnetic materials under an external magnetic field. Once the magnetic field is removed, their average magnetization becomes zero due to the random flipping of particles' magnetic moments (Issa et al., 2013; Kolhatkar et al., 2013).

References

Akbarzadeh, A., Samiei, M., and Davaran, S. (2012). Magnetic nanoparticles : preparation , physical properties , and applications in biomedicine. *Nanoscale Research Letters* 7(1)**,** 144-144. doi: 10.1186/1556-276X-7-144.

Bean, C.P., and Livingston, J.D. (1959). Superparamagnetism. *Journal of Applied Physics* 30(4)**,** S120-S129. doi: 10.1063/1.2185850.

Bhuvanendran Nair Gourikutty, S., Chang, C.P., and Puiu, P.D. (2016). Microfluidic immunomagnetic cell separation from whole blood. *J Chromatogr B Analyt Technol Biomed Life Sci* 1011**,** 77-88. doi: 10.1016/j.jchromb.2015.12.016.

Brown, W.F. (1963). Thermal Fluctuations of a Single-Domain Particle. *Physical Review* 130(5)**,** 1677-1686.

Durmus, N.G., Tekin, H.C., Guven, S., Sridhar, K., Yildiz, A.A., Calibasi, G., et al. (2015). Magnetic levitation of single cells. *Proceedings of the National Academy of Sciences* 112(28)**,** E3661-E3668.

Gerber, R., Takayasu, M., and Friedlaender, F.J. (1983). *Generalization of HGMS Theory: The Capture of Ultra-fine Particles.*

Gijs, M.A.M. (2004). Magnetic bead handling on-chip: New opportunities for analytical applications. *Microfluidics and Nanofluidics* 1(1)**,** 22-40. doi: 10.1007/s10404-004-0010-y.

Gijs, M.A.M., Lacharme, F.d.r., and Lehmann, U. (2010). Microfluidic applications of magnetic particles for biological analysis and catalysis. *Chemical Reviews* 110(3)**,** 1518-1563. doi: 10.1021/cr9001929.

Hejazian, M., Li, W., and Nguyen, N.-T. (2015). Lab on a chip for continuous-flow magnetic cell separation. *Lab on a Chip* 15(4)**,** 959-970. doi: 10.1039/C4LC01422G.

Issa, B., Obaidat, I.M., Albiss, B.A., and Haik, Y. (2013). Magnetic nanoparticles: Surface effects and properties related to biomedicine applications. *International Journal of Molecular Sciences* 14(11)**,** 21266-21305. doi: 10.3390/ijms141121266.

Kolhatkar, A.G., Jamison, A.C., Litvinov, D., Willson, R.C., and Lee, T.R. (2013). *Tuning the magnetic properties of nanoparticles.*

Pamme, N. (2006). Magnetism and microfluidics. *Lab on a chip* 6(1)**,** 24-38. doi: 10.1039/b513005k.

Pankhurst, Q.A., Connolly, J., Jones, S., and Dobson, J. (2003). Applications of magnetic nanoparticles in biomedicine. *Journal of physics D: Applied physics* 36(13)**,** R167.

Sun, C., Lee, J.S.H., and Zhang, M. (2008). Magnetic nanoparticles in MR imaging and drug delivery. *Advanced Drug Delivery Reviews* 60(11)**,** 1252-1265. doi: 10.1016/j.addr.2008.03.018.

Weston, M.C., Gerner, M.D., and Fritsch, I. (2010). Magnetic Fields for Fluid Motion. *Analytical Chemistry* 82(9)**,** 3411-3418. doi: 10.1021/ac901783n.
